# Computational mapping of resistance-relevant signals in proteins using deep and classical feature spaces

**DOI:** 10.3389/fmicb.2026.1899081

**Published:** 2026-08-19

**Authors:** Rahul Kaushik, Suyong Re

**Affiliations:** Laboratory of In-silico Design, Artificial Intelligence Center for Health and Biomedical Research, National Institutes of Biomedical Innovation, Health and Nutrition (NIBN), Settsu, Osaka, Japan

**Keywords:** alignment-free functional annotation, antimicrobial resistance proteins, macromolecular signatures, physicochemical descriptors, protein language models, sequence-function relationship

## Abstract

**Introduction:**

The functional diversity of antimicrobial resistance (AMR) proteins necessitates advanced computational frameworks capable of capturing complex macromolecular signatures beyond simple sequence homology. While traditional alignment-based tools identify known resistance determinants, they often overlook the underlying biochemical and structural properties that define novel or divergent resistant macromolecules.

**Methods:**

This study systematically investigates the capacity of classical sequence-derived descriptors and deep protein language model (pLM) embeddings to resolve these essential functional characteristics at the protein level and contribute to predictive robustness and generalization. Multiple machine learning classifiers were trained using classical features, deep embeddings, and their integrated representations to assess the consistency of AMR-related signal detection.

**Results and discussion:**

Across models, both feature types demonstrated strong discriminative capacity while deep embeddings-based detection outperforming the classical features-based detection. Further, their integration did not produce substantial performance gains over deep embeddings alone, however it consistently improved stability and generalization across classifiers and validation settings. These findings indicate that classical descriptors encode resistance-relevant signals that complement contextual representations learned by protein language models. The AUROC values approached 0.85 for classical features alone, 0.97 for deep embeddings alone and 0.94 for integrated feature space across experimental configurations, confirming that alignment-free numerical representations effectively capture AMR-associated functional characteristics. Benchmarking against diverse AMR identification tools further demonstrates that classical and deep representations-based approaches maintain sensitivity toward divergent sequences while preserving high specificity, individually as well as collectively. All datasets, models, and scripts are publicly available at https://github.com/DrRahulKaushik/ProARG.

## 1 Introduction

Antimicrobial resistance (AMR) is a major global health threat that compromises the treatment of bacterial infections and undermines modern medical practice. Extensive antibiotic use across clinical, veterinary, agricultural, and environmental settings has intensified selective pressure on microbial populations, accelerating the emergence and dissemination of resistant pathogens (Jean et al., 2022; Murray et al., 2022; Naghavi et al., 2024; Bhattacharya et al., 2021; Kalita et al., 2019). Infections once readily treatable now exhibit increased persistence and mortality, imposing substantial clinical and economic burdens worldwide (Tewolde et al., 2025; Thombre et al., 2019; Kant et al., 2024). International health agencies warn that unchecked AMR could reverse decades of progress in infectious disease control (Kaushik and Re, 2026; Darby et al., 2023). At the cellular level, AMR arises through molecular strategies that enable bacteria to survive antibiotic exposure. These include enzymatic drug inactivation, target modification, reduced membrane permeability, active efflux, and metabolic bypass mechanisms (Darby et al., 2023; Alem et al., 2025; Eisenreich et al., 2022). Resistance determinants may be intrinsic or acquired via horizontal gene transfer mediated by plasmids, transposons, and integrons, facilitating rapid spread across species and ecological niches (Kaushik and Re, 2026; Alem et al., 2025; Smith et al., 2022). High-throughput sequencing has revealed a vast and largely uncharacterized resistome spanning clinical, commensal, and environmental microbiota (Eisenreich et al., 2022; Wang et al., 2022; Muñoz et al., 2024). However, converting this expanding sequence space into clinically actionable insight remains challenging.

Current AMR detection approaches rely primarily on phenotypic susceptibility testing or sequence similarity–based annotation. Phenotypic assays provide direct functional evidence but are time-consuming and may delay treatment (Darby et al., 2023; Wang et al., 2022; Li et al., 2024). Sequence-based tools align query proteins against curated resistance databases, enabling rapid annotation but depending heavily on similarity thresholds and reference coverage (Gschwind et al., 2023; Alcock et al., 2023; Feldgarden et al., 2021). While effective for known genes, these methods often miss divergent variants or novel resistance determinants lacking close homologs (Alcock et al., 2023; Feldgarden et al., 2021; Feldgarden et al., 2022). Moreover, alignment-based approaches do not explicitly model distributed sequence patterns or higher-order dependencies that may encode resistance phenotypes. Machine learning (ML) provides an alternative by learning discriminative patterns directly from sequence data (Abramson et al., 2024; Mirdita et al., 2022; Kaushik and Zhang, 2022a, b). ML-based approaches have been applied to resistance phenotype prediction, gene function inference, and antimicrobial target prioritization (Bilal et al., 2025; Shanker et al., 2024; Szymczak et al., 2025; Santos-Júnior et al., 2024). More recently, transformer-based protein language models trained on large sequence corpora have demonstrated the capacity to capture structural, functional, and evolutionary constraints from primary amino acid sequences alone (Abramson et al., 2024; Mirdita et al., 2022; Hayes et al., 2025; Lin et al., 2023; Kaushik et al., 2023). These embeddings offer an alignment-free representation that complements classical descriptors such as amino acid composition and physicochemical features (Arango-Argoty et al., 2018; Frazer et al., 2021; Kaushik and Zhang, 2020; Mall et al., 2025).

**Figure 1:**
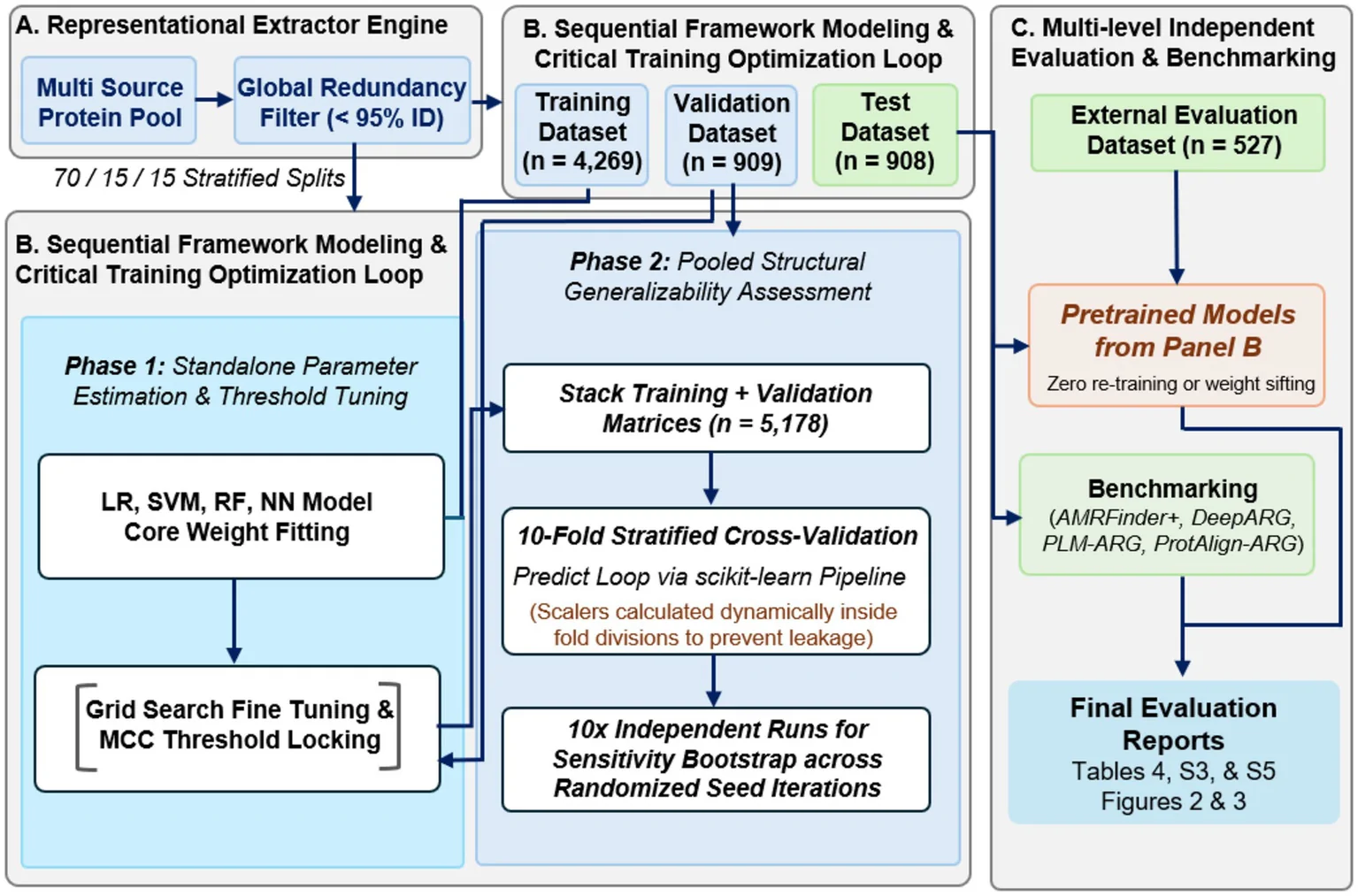
The schematic outlines the systematic pipeline for high-confidence dataset curation, dual-stream feature engineering integrating classical descriptors with deep protein language model embeddings, stratified model training and optimization, and performance benchmarking. Panel **(A)** illustrates the global consolidation of the protein pool, followed by CD-HIT pre-clustering at a strict 95% sequence identity threshold to eliminate cross-partition redundancy before undergoing a stratified 70/15/15 isolation split. Panel **(B)** outlines the two-phase training strategy. Phase 1 details model core weights fitting on the Training dataset (*n* = 4,269) coupled with intermediate hyperparameter tuning and decision-threshold optimization using the Validation dataset (*n* = 909). Phase 2 details the pooling of these sets (*n* = 5,178) for a 10-fold stratified cross-validation predict loop executed inside isolated scikit-learn Pipeline objects to prevent preprocessing leakage, evaluated across 10 independent randomized seed iterations. Panel **(C)** depicts the evaluation protocol where the locked, pre-saved trained models are tested on the hidden internal Test dataset (*n* = 908) and a completely external, novel *β*-lactamase surveillance cohort (*n* = 527) without any re-training or weight adjustments.

Despite substantial advances in computational AMR detection, systematic and controlled comparisons between classical sequence-derived descriptors and deep protein language model embeddings remain limited, particularly with respect to their complementary contributions to generalization. To address these issues, we implement a rigorously curated, protein-level, alignment-free evaluation framework for AMR detection. The emphasis of this study is not on architectural novelty, but on representation analysis, dataset quality, redundancy control, and transparent evaluation. We examine two complementary feature spaces: (i) classical compositional and physicochemical descriptors derived directly from primary sequence and (ii) contextual embeddings generated by transformer-based protein language models. These representations are evaluated independently and in integrated form across multiple supervised classifiers using stratified validation and balanced performance metrics. This systematic assessment clarifies the relative and combined contributions of classical and deep embeddings to AMR signal capture. While deep embeddings provide strong standalone performance, the integration of classical descriptors contributes to robustness and stability across models and validation settings. The resulting alignment-free framework evaluation offers a scalable strategy for detecting both canonical and sequence-divergent AMR determinants and establishes a principled foundation for future incorporation of genomic context, structural signals, and mechanism-level stratification. A schematic outlines the systematic pipeline for high-confidence dataset curation, dual-stream feature engineering integrating classical descriptors with deep protein language model embeddings, stratified model training and optimization, and comprehensive performance evaluation is depicted in Figure 1.

## 2 Materials and methods

### 2.1 Training and testing data compilation

This study establishes a proof-of-concept artificial intelligence and machine learning (AI/ML) framework for detecting antimicrobial resistance (AMR) directly from prokaryotic protein sequences. We compiled a high-confidence, sequence-only dataset of AMR-positive and AMR-negative proteins from curated public resources while minimizing redundancy, annotation noise, and label leakage. Only experimentally curated or reference-quality datasets were included to ensure biological validity.

#### 2.1.1 AMR-positive and AMR-negative sequence datasets construction

AMR-associated proteins were collected from three established databases. The Comprehensive Antibiotic Resistance Database (CARD) (Alcock et al., 2023) served as the primary reference source and contributed a dataset of 6,052 protein sequences. To increase annotation diversity and reduce dependence on a single resource, AMRFinderPlus (Feldgarden et al., 2022) reference datasets were incorporated, including manually curated protein sequences (*n* = 9,862) linked to known resistance phenotypes. ResFinder (Gschwind et al., 2023) was also included to broaden representation of clinically relevant acquired resistance genes. The consolidated ResFinder FASTA file (*n* = 3,212) was used; class-specific sequence files were excluded to prevent duplication.

During quality control filtering, sequences ≥50 amino acids containing only standard residues were retained. After filtering, all AMR proteins were merged. Redundancy was reduced by using CD-HIT (Huang et al., 2010) clustering at 95% sequence identity to remove near-identical sequences while preserving functional diversity. The final non-redundant AMR-positive dataset comprised 3,043 proteins. AMR-negative proteins were obtained from NCBI RefSeq bacterial protein releases (Goldfarb et al., 2025) to ensure broad phylogenetic coverage. Genome-derived proteins ≥50 amino acids with standard residues were retained. To minimize latent AMR protein contamination, all RefSeq proteins were screened against the curated AMR-positive dataset. Sequences were excluded if they met ≥30% identity, ≥50% query coverage, and E-value ≤1e-5 against any AMR-positive protein. The remaining 49,001 proteins were designated high-confidence AMR-negative candidates.

#### 2.1.2 Dataset balancing and partitioning

Because the AMR-negative dataset significantly exceeded the AMR-positive set, random under-sampling without replacement was applied to select 3,043 AMR-negative proteins, matching the AMR-positive count. The final balanced dataset contained 6,086 proteins (3,043 AMR-positive labeled 0; 3,043 non-AMR labeled 1). The sequences were partitioned at the sequence level into training (70%; 4,269 proteins comprising 2,135 AMR-positive and 2,134 AMR-negative proteins), validation (15%; 909 proteins comprising 457 AMR-positive and 452 AMR-negative proteins), and test (15%; 908 proteins comprising 451 AMR-positive and 457 AMR-negative proteins) sub-datasets, ensuring no identical or near-identical sequences appeared across splits.

The sequence similarity between dataset partitions was assessed using an all-vs-all pairwise alignment strategy across partitions. Specifically, pairwise sequence identities were computed for Training vs. Validation to ensure unbiased hyper-parameter optimization, Training vs. Test, and Validation vs. Test protein datasets to ensure transparent evaluation of feature spaces. For each comparison, every sequence in one partition was aligned against all sequences in the other partition to identify the closest match and the resulting pairwise percentage identity for the topmost match was considered for the alignment results. The mean identity values were derived from these cross-partition alignment values. A pairwise sequence identity analysis further supported non-redundancy between partitions. The average percentage identity between the Training and Validation datasets was 43.9 ± 18.53, between the Training and Test datasets 42.8 ± 17.80, and between the Validation and Test datasets 37.1 ± 12.85, indicating substantial sequence divergence across splits. The test dataset remained completely independent and was only used once for final evaluation. The training dataset was used for model training and 10-fold cross validation. While a separate independent validation dataset served the purpose of hyper-parameter optimization. Partition-specific protein sequence files were retained to allow independent regeneration of features and embeddings. An overview of dataset construction for AMR-positive and AMR-negative related proteins and the preprocessing of the constructed dataset in this study is provided in Table 1.

**Table 1:** An overview of dataset construction for AMR and non-AMR related proteins and the preprocessing of the constructed dataset for model training.

Step	Input data	Method/key parameters	Output
Sequence curation	AMR and non-AMR protein	Source-level label assignment; duplicate removal	Curated protein dataset
Redundancy check	Curated protein sequences	Sequence clustering at 95% identity	Non-redundant protein set
Dataset partitioning	Labeled feature matrix	Stratified split (70% train, 15% validation, 15% test)	Partitioned feature datasets
Classical features	Clustered protein sequences (training)	AAC, dipeptide, tripeptide, and physicochemical descriptors	Classical feature matrix
Deep embeddings	Protein FASTA (training)	Pretrained ESM-2 model; mean pooling; no fine-tuning	Dense embedding matrix
Feature Integration	Classical + deep features (training)	Feature-wise concatenation without imputation	Integrated feature matrix
Label attachment	Integrated features (training)	Deterministic mapping from sequence identifiers	Labeled dataset
Quality control	Partitioned datasets	Label balance checks, leakage tests, random-label baseline	Validated datasets

### 2.2 Feature extraction from protein sequences

Two complementary representation strategies were used to evaluate AMR detection from primary sequence information alone: classical sequence-derived features and deep protein embeddings.

#### 2.2.1 Classical sequence-based features

Proteins were encoded into fixed-length vectors using established descriptors. Four feature categories were extracted: (i) amino acid composition, normalized frequencies of 20 residues; (ii) dipeptide composition, frequencies of 400 adjacent residue pairs; (iii) tripeptide composition, frequencies of 8,000 adjacent triplets; and (iv) physicochemical properties, including hydrophobicity, charge, polarity, molecular weight, and aromaticity. In total, 8,435 features were generated per protein. Physicochemical properties were encoded as global sequence averages and aggregated values over sliding dipeptide and tripeptide windows. All features were standardized using z-score normalization prior to training.

#### 2.2.2 Deep protein embedding features

Deep embeddings were generated using Evolutionary Scale Modeling (ESM) (Hayes et al., 2025; Lin et al., 2023), a pretrained transformer-based protein language model trained on large-scale sequence corpora. ESM models learn contextual amino acid representations that capture structural, functional, and evolutionary information without alignment or external annotation. Protein sequences were processed using a pretrained ESM-2 model with fixed weights without any fine-tuning. The model produced high-dimensional residue-level embeddings for each amino acid. Residue embeddings were aggregated via mean pooling to generate a single 1,280-dimensional protein-level vector per sequence. Embeddings were stored in tabular format and used independently or concatenated with classical features. Feature integration occurred only after embedding generation and verification, and class labels were appended at the final stage to prevent label leakage.

### 2.3 Stratified model training and validation

Four supervised classifiers representing distinct learning paradigms were evaluated: Logistic Regression (linear), Support Vector Machine (kernel-based), Random Forest (ensemble), and a feed-forward Neural Network. All models were trained and 10-fold cross validated using the Training dataset (*n* = 4,269 proteins), while hyperparameter optimization and decision threshold tuning was performed using the Validation dataset (*n* = 909 proteins). Following this initial phase, the training and validation datasets were combined to conduct a comprehensive 10-fold cross-validation to assess overall model stability. The trained and optimized model were evaluated on the independent Test dataset (*n* = 908 protein). Details of these classifiers are provided in Supplementary material (Note 1). All preprocessing steps, including feature scaling, model fitting, and hyperparameter selection, were performed exclusively on Training and Validation datasets. A 10-Fold cross validation was performed on the pooled training-validation space to verify structural generalization. Random seeds were fixed during dataset splitting and model initialization to ensure reproducibility. Additionally, to evaluate sensitivity and ensure robustness against random initialization bias, training and optimization were repeated across 10 independent iterations using completely unique random seeds, and performance was evaluated on the independent Test dataset.

To eliminate any potential data-partition bias and confirm that the final evaluation metrics are independent of any single, static data split, a repeated randomized resampling framework was implemented using 50 distinct iterations. For each randomized iteration, the compiled dataset was merged and dynamically re-partitioned into a frozen 70:15:15 training, validation, and out-of-sample testing split. All scaling parameters, feature mappings, and classifier fits were computed strictly and independently within each localized training split. Model decision thresholds were dynamically optimized on the validation split using Matthews Correlation Coefficient (MCC) maximization, and final out-of-sample generalization metrics were gathered exclusively from the untouched test split. Aggregate performance values across the 50 shuffles were compiled as an empirical mean ± standard deviation (SD) for the LR, SVM, RF, NN, and FM architectures, ensuring that the performance ceilings reflect structurally stable characteristics of the biophysical descriptor space.

Hyperparameters were selected using performance evaluation on Validation dataset. The Logistic Regression implemented L2 regularization having fixed inverse regularization strength, serving as a stable linear baseline in high-dimensional space. The Support Vector Machine implemented radial basis function kernel with validated regularization and kernel parameters optimized via grid search to model non-linear decision boundaries. The Random Forest classifier implemented ensemble size and feature subsampling configured to ensure convergence and robustness to noise. The Neural Network classifier implemented feed-forward architecture with ReLU activations, dropout regularization, the Adam optimizer, and early stopping based on validation loss. Additional details of these methods are provided in Supplementary material (Note). Class weighting was applied where appropriate to ensure balanced penalization of misclassification errors. Default library parameters were retained when validation performance was strong, reducing overfitting risk. Complete architectural and hyperparameter details are summarized in Table 2.

**Table 2:** A summary of hyperparameters used for machine learning models.

Model	Hyperparameter	Rationale
Logistic Regression	Penalty: L2	Prevents overfitting in high-dimensional space
	Solver: saga	Efficient classification for large feature sets
	C: 1.0	Inverse regularization strength
	Max iterations: 5000	Ensures convergence in high-dimensional space
	Scaling: standard scaler	Required for stable gradient optimization
Support Vector Machine (SVM)	Kernel: RBF	Captures non-linear decision boundaries
	C: 0.1, 1, 10, grid search	Regularization parameter control margin width
	Gamma: scale, 0.01, 0.001	Controls kernel width
	Class weight: balanced	Equal penalization of misclassification
	Probability: enabled	Required for AUROC computation
Random Forest	Trees: 500	Ensures stable ensemble predictions
	Criterion: gini impurity	Measures node purity
	Min samples per leaf: 2	Allows fine-grained splits
	Max feature: sqrt	Reduces correlation between trees
	Max depth: none	Allows full tree growth
Neural Network	Architecture: 512–256	Fully connected feed-forward network
	Activation: ReLU	Efficient gradient propagation
	Alpha(L2): 1e-4	Regularization to reduce overfitting
	Early stopping: enabled	Prevents overfitting (validation fraction = 0.1)
	Max iterations: 500	Ensures convergence in high-dimensional space
	Optimizer: Adam	Adaptive gradient optimization

Random seed 42 and an optimized MCC threshold was used for training of all the models to ensure reproducibility and balanced classification, respectively.

### 2.4 Negative control: shuffled-label and scrambled-sequence frameworks

To empirically verify that the predictive models are capturing genuine structural, evolutionary, and contextual biological signals rather than exploiting hidden dataset artifacts or multi-collinear biases, a two-level randomized negative control evaluation was integrated into the validation pipeline.

The first level of control comprised a Shuffled-Label check to test class-association thresholds. The target label array (y) was decoupled from the standardized feature matrix (X) and subjected to randomized global permutation (np.random.permutation). The second level of control comprised a Scrambled-Sequence analysis to verify sequence-level dependency and language model contextual sensitivity. For every protein in the study, the primary sequence string was converted into a mutable vector, stripped of trailing termination tokens, and randomized using a multi-tiered structural disruption script. To systematically evaluate the models’ dependency on local biological grammar, sequence scrambling was executed at three progressive topological tiers: single residue positions (monopeptide level), pairs of adjacent residues (dipeptide level), and blocks of three sequential residues (tripeptide level). For each protein sequence I the independent Test dataset (*n* = 908), a localized window-shuffling algorithm was applied to shuffle a minimum of 10% of the total residue positions at once while strictly maintaining the native class label. This stratified approach selectively destroys localized motifs, functional domains, k-mer distributions, and transformer contextual paths at varying scales of complexity. Crucially, it leaves macro-compositional properties, specifically exact sequence lengths and global amino acid single counts, entirely invariant. These scrambled strings were re-written into a control FASTA cohort and passed through our primary feature extraction pipeline to generate a fresh, matching space of 9,715 classical and deep ESM-2 features. This control matrix was standardized using the pre-saved pipeline scaler coefficients (joblib.load) and evaluated directly against our pre-trained, locked classifier configurations to record metrics for uninformative sequence states.

### 2.5 External curated validation dataset

To evaluate the generalizability of the pre-trained classification models and ensure that the learned numerical representations are invariant to primary dataset boundaries, an external validation dataset was curated (*n* = 527 protein sequences). The antimicrobial resistance (AMR) positive cohort comprised 272 highly mutable, clinically critical TEM, SHV, and CTX-M *β*-lactamase enzymes. To establish a rigorous negative control baseline, these resistance sequences were balanced against 255 non-AMR bacterial protein sequences systematically fetched from the NCBI RefSeq database. The non-AMR protein proteins were extracted from 49,001 proteins as compiled in Section 2.1.1. These were completely exclusive of the 3,043 non-AMR dataset mentioned in Section 2.1.2. This external cohort (*n* = 527) was subjected to identical sequence processing pipelines but was kept strictly insulated from all upstream feature extraction profiling, scaling fitting, cross-validation partitioning, or hyperparameter selection stages, serving as an absolute blind test for universal generalizability.”

### 2.6 Model evaluation metrics

To ensure comprehensive and class-balanced assessment, five complementary metrics were used. (i) Area Under the Receiver Operating Characteristic Curve (AUROC): A threshold-independent metric assessing discrimination between AMR-positive and AMR-negative proteins. Values range from 0.5 (random) to 1.0 (perfect); (ii) Matthews Correlation Coefficient (MCC): A balanced metric incorporating true positives, true negatives, false positives, and false negatives. MCC ranges from −1 to 1 and remains informative under class imbalance; (iii) F1-Score: Harmonic mean of precision and recall, quantifying the balance between sensitivity and specificity, particularly relevant when both false positives and false negatives are consequential; (iv) Precision: Measures the accuracy of positive predictions, while minimizing false positives; and (v) Recall: quantifies the efficiency to find all positive instances, while minimizing false negatives. Together, these metrics provide a non-redundant robust evaluation of discrimination capacity and practical prediction reliability.

### 2.7 Computational resources and availability

All experiments were conducted on a high-performance computing (HPC) environment with CPU and GPU nodes with fixed random seeds. All the scripts, datasets, and prediction models are made publicly available through GitHub at https://github.com/DrRahulKaushik/ProARG.

## 3 Results

### 3.1 Feature space characteristics and signal analysis

#### 3.1.1 Feature inventory and dimensionality

The integrated feature space consisted of 9,715 quantitative descriptors per protein, including 8,435 classical and 1,280 deep-embeddings based representations. To characterize the effective dimensionality of different feature representations, principal component analysis (PCA) was applied independently to the classical sequence-derived features, deep embedding features, and their integrated combination. To ensure maximum statistical validity and prevent arbitrary scale distortions from biasing the variance metrics, PCA configurations were evaluated under both raw unscaled and standardized (z-score normalized) conditions.

Under raw unscaled conditions, classical sequence-derived descriptors showed extremely low intrinsic dimensionality, with a single principal component explaining more than 95% of the total variance. This pattern indicates that raw variance across many compositional and physicochemical descriptors is dominated by a small number of global trends affecting large groups of features simultaneously, primarily driven by numeric scale inequalities in bulk sequence properties. However, when proper standardization was enforced to equalize feature scale contributions, the true multivariate complexity emerged: 750 principal components were required to explain 50% of the variance, 1,934 components for 80%, and 3,436 components to capture 95% of the total variance. This wide dispersion mathematically demonstrates a highly multi-collinear and complex underlying topology. To visually map how these distinct information topologies behave across varying scales of variance complexity, we introduced a 3 × 3 multi-panel filtered subspace reconstruction matrix (Supplementary Figure S1) that isolates the principal coordinate space at the exact 50, 80, and 95% cumulative variance boundaries before generating a localized, re-projected mapping of PC1 against PC2. In the Classical features, tracking the data up to the 95% variance boundary requires 3,436 components, where its local PC1 and PC2 capture a minute fraction of the subspace variance (0.7 and 0.4%, respectively). This extreme variance diffusion confirms a highly complex topology where predictive signals are scattered across thousands of axes, mathematically explaining why simple linear models fail and non-linear classifiers are required.

In contrast, deep embedding features exhibited substantially higher dimensional complexity under basic scaling profiles but maintained an inherently structured information architecture when normalized. Four principal components were required to explain 50% of the features variance, 42 components for 80%, and 244 components to capture 95% of the total features variance in unscaled space. Upon applying standard standardization, the structural metrics stabilized efficiently, requiring 18 components for 50%, 90 components for 80%, and 362 components to reach the 95% cumulative variance threshold. This behavior reflects the richer yet highly optimized representational capacity of transformer-derived embeddings, which encode diverse contextual and evolutionary sequence signals across multiple relatively independent dimensions that have been pre-computed and orthogonalized by the language model layers. As illustrated in Supplementary Figure S1, the Deep ESM Embedding row concentrates information much more efficiently than classical feature arrays. At the 95% variance checkpoint (encompassing 362 components), its localized PC1 and PC2 retain a high share of variance (9.1 and 6.7%, respectively). This represents a 14-fold concentration of information density along the primary axes compared to classical features, visually and mathematically demonstrating that the transformer layers group complex properties into a structured latent space that directly justifies why simple linear baselines like Logistic Regression achieve high generalizability within the pure deep embedding space.

When classical and deep features were combined into a single integrated representation, the effective unscaled dimensionality again appeared highly compressed, with one principal component accounting for more than 95% of the total variance. This outcome indicates that large-scale variance trends and raw scalar disparities shared across feature types dominate the overall variance structure of the integrated feature space prior to scaling, even though substantial fine-scale variability remains distributed across many dimensions. When standardized, the integrated space matched the decentralized dispersion of the classical space, requiring 658 components for 50%, 1,869 components for 80%, and 3,432 components to capture 95% of the total cumulative variance. This structural dispersion confirms that simple concatenation introduces extreme information diffusion and multi-collinearity, wrapping the class boundaries in a highly convoluted space that mathematically necessitates non-linear kernels (such as RBF SVMs or Neural Networks) to map effectively. This dispersion is further evidenced in the Integrated row of Supplementary Figure S1, where the localized PC1 and PC2 at the 95% variance checkpoint are diluted down to capturing just 1.7 and 1.1% of the subspace variance, respectively. Supplementary Table S1 summarizes the structural and discriminative properties of classical, deep, and integrated feature spaces.

#### 3.1.2 Feature value distributions

For each feature, the mean value in the AMR-positive and AMR-negative class was computed, and the absolute difference between class means was used as a measure of effect size. Out of the total feature set, 5,251 features exhibited higher mean values in AMR-positive proteins, while 4,463 features showed higher mean values in AMR-negative proteins, indicating broadly distributed shifts rather than unidirectional scaling differences. The mean absolute difference between class means was 0.971, while the median absolute difference was close to zero (2.51e-5), reflecting a skewed distribution in which a subset of features shows strong class-specific shifts, whereas many features contribute weakly or redundantly.

To formally assess statistical separability, non-parametric Mann–Whitney U tests were performed for all features with Benjamini-Hochberg false discovery rate (FDR) correction. Of the 9,715 features tested, 4,216 (43.4%) features were significantly different at FDR < 0.05, 3,267 (33.6%) at FDR < 0.01, and 2,438 (25.1%) at FDR < 0.001, demonstrating that a substantial fraction of the feature space carries statistically meaningful class-discriminative signals (Supplementary Table S1). The median FDR value across all features was 0.0506, indicating that while not all features are individually informative, a large subset exhibits strong class association. Crucially, this extensive footprint of individual feature significance, juxtaposed against the high multivariate multi-collinearity detailed in Section 3.1.1, empirically solidifies why linear modeling baselines see massive generalizability leaps in the pre-optimized embedding space but fail in the diffused classical space.

#### 3.1.3 Classical vs. deep feature contribution

To further characterize relationships among features, redundancy within each feature category was quantified using mean absolute pairwise correlation. This metric captures local linear associations between individual feature pairs and provides a complementary view of feature dependence that differs from variance-based dimensionality measures such as PCA.

Classical features displayed very low average pairwise correlation (mean absolute correlation = 0.013). Although unscaled PCA revealed strong variance concentration in the first principal component, this does not necessarily imply high pairwise correlation across all individual features. Instead, it suggests that many features respond similarly to a small number of underlying variance patterns while remaining only weakly correlated on a pairwise basis. However, as detailed in Section 3.1.1, when standard standardization is applied, this space requires 3,436 independent principal components to capture 95% of the cumulative variance. This juxtaposition proves that while classical features lack tight pairwise dependencies, they form a massive, highly diffused multivariate topology. This structural dispersion scatters predictive signals across thousands of axes, wrapping class boundaries in a highly complex and non-linear geometry.

Deep embedding features showed moderately higher redundancy as compared to classical features (mean absolute correlation = 0.119), reflecting shared contextual and evolutionary information learned by the pretrained transformer model. Because embedding vectors are generated through a common representation learning process, moderate correlations among dimensions are expected. Crucially, this moderate pairwise correlation aligns with a highly compressed and optimized multivariate structure when standardized, requiring only 362 principal components to capture 95% of the total variance. The transformer layers have effectively pre-computed these spatial relationships, uncoupling raw sequence dependencies and grouping the biological signals into a compact latent coordinate system.

The integrated feature set showed low redundancy overall (mean absolute correlation = 0.017), comparable to classical features and lower than deep embeddings alone. This indicates that combining classical descriptors with embeddings introduces complementary information while reducing correlated structure within the embedding space. Together with the PCA results, these observations suggest that the integrated representation captures dominant variance trends while preserving a diverse set of relatively independent predictive signals. Nonetheless, because the integrated space inherits the massive, decentralized dimensionality of the classical descriptors (requiring 3,432 components for 95% standardized variance), the pure embedding coordinates become diluted. This structural swamping increases overall multi-collinear noise and creates a convoluted classification hyper-plane, which explains why the integrated feature set requires complex non-linear kernels to resolve and ultimately scores lower than the standalone deep embedding models.

#### 3.1.4 Feature separability and signal assessment

To exclude the possibility that apparent separability arose from dataset artifacts or information leakage, a randomized-label control analysis was performed. When class labels were permuted, the projected feature distributions showed complete overlap, and classification performance collapsed to near-random levels (AUROC = 0.492), approximating random expectation of 0.50. Furthermore, to evaluate whether the models were capturing biologically meaningful signals or merely responding to simple amino acid composition, we implemented a sequence-scrambling control. For this, all sequences in the Test set were randomly scrambled to destroy their primary structure and functional motifs while maintaining their original amino acid frequencies. The scrambling was performed at one residue (single residue position shuffling), followed by two residues (dipeptide level sequence shuffling), and finally three residues (tripeptide level sequence shuffling) levels for 10% residue positions of an individual sequence without affecting the sequence label. Evaluation on this scrambled dataset resulted in a sharp decline in predictive power. The MCC for all models collapsed to near-random levels (0.079 ≤ MCC ≤ 0.193), and AUROC values decreased significantly (0.57 ≤ AUROC ≤ 0.65) compared to the high performance observed on the true biological sequences. The results of sequence-scrambling control experiment are summarized in Table 3. Notably, the Random Forest model had a very high Recall (0.99) but a very low MCC (0.07). This indicates that on the scrambled dataset, the RF model is simply guessing the positive class for almost everything, reflecting that the model’s logic breaks down without the proper sequence order. This divergence confirms that the structural and contextual information encoded in our feature space, particularly via the deep learning-based embeddings, is essential for accurate classification and that the model is indeed learning relevant biological features.

**Table 3:** Evaluation of model performance on a sequence-shuffled (scrambled) null control derived from the independent test dataset (*n* = 908).

Model	AUROC	MCC	F1	Precision	Recall
Logistic Regression	0.571	0.108	0.618	0.541	0.720
Support Vector Machine	0.639	0.193	0.663	0.564	0.805
Random Forest	0.654	0.079	0.673	0.508	0.993
Neural Network	0.622	0.104	0.655	0.527	0.862
Fusion Model	0.641	0.097	0.675	0.536	0.872

The scrambling was performed at one residue (single residues position shuffling), followed by two residues (dipeptide level sequence shuffling), and finally three residues (tripeptide level sequence shuffling) levels for 10% residue positions of an individual sequence without affecting the sequence label.

### 3.2 Model performance and comparative evaluation

The predictive capability through different features spaces, individually and collectively, was evaluated by implementing four machine learning based classification models, viz. Logistic Regression (LR), Support Vector Machine (SVM), Random Forest (RF), and a feed-forward Neural Network (NN), on 10-Fold cross validation during training and on an independent Validation dataset. Additionally, a Fusion Model (FM) by integrating (stacking) LR, SVM, RF, and NN was also trained and evaluated.

#### 3.2.1 Effect of feature representation on predictive performance

**Figure 2:**
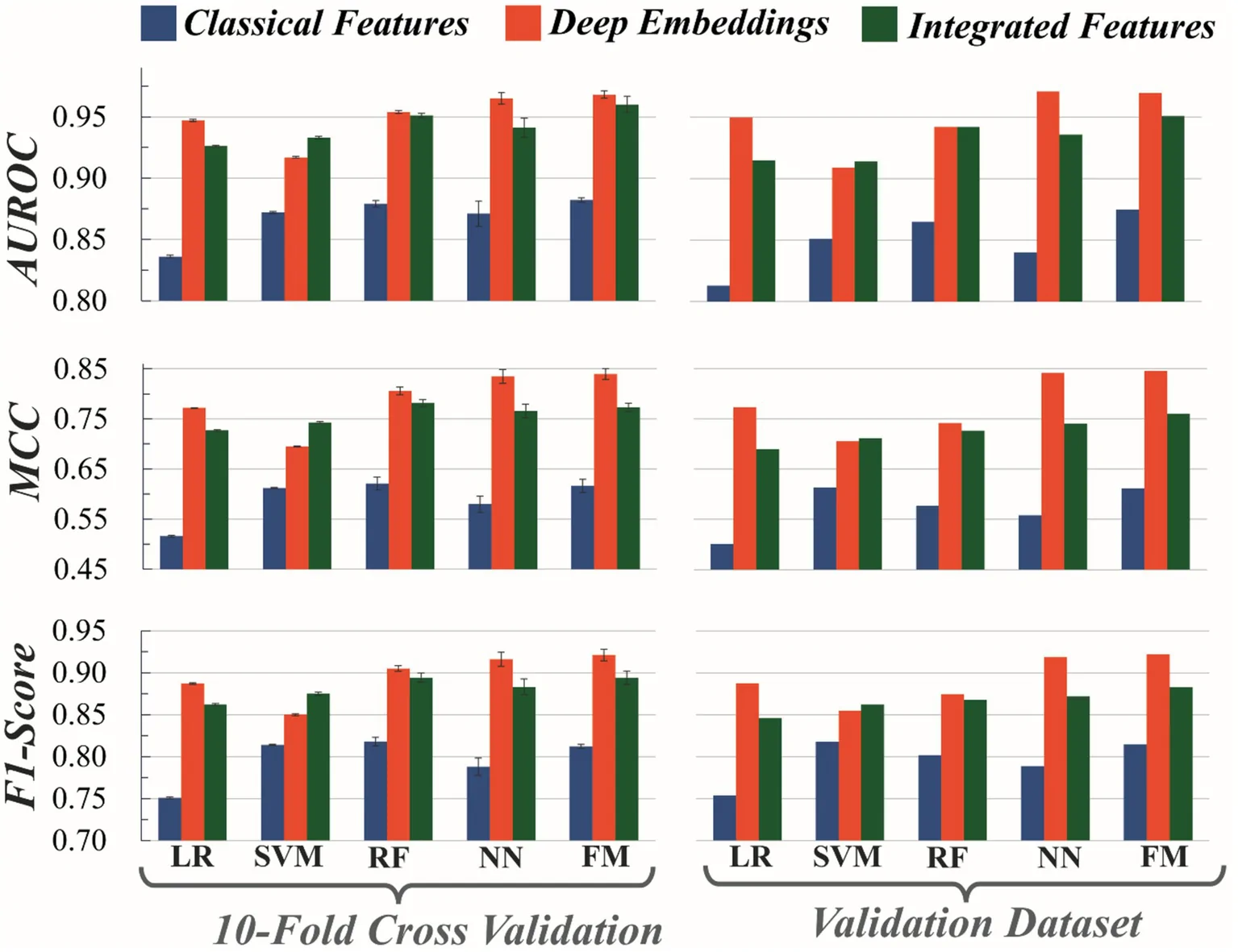
Performance of machine learning models evaluated through 10-fold cross validation on training dataset (*n* = 4,269) and independent validation dataset (*n* = 909). Validation dataset evaluation metrics were used for hyperparameter optimization. AUROC denotes the area under the receiver operating characteristic curve, MCC denotes Matthews’ correlation coefficient, and F1-Score represents the harmonic mean of precision and recall. LR, Logistic Regression; SVM, Support Vector Machine; RF, Random Forest; NN, Neural Network; FM, Fusion Model.

To quantify the impact of different representational strategies on antimicrobial resistance (AMR) detection, we systematically compared the performance of models trained on three distinct feature sets: classical features, deep embeddings, and integrated features. This evaluation was conducted across four diverse machine learning architectures and a fusion model to ensure that observed performance gains were attributable to the representation rather than specific model biases. The results for the 10-fold cross validation during training and evaluation on the independent Validation dataset are summarized in Supplementary Table S2 and depicted in Figure 2 (with corresponding held-out Test dataset performance detailed in Supplementary Table S3).

##### 3.2.1.1 Performance of classical sequence-derived features

Models trained exclusively on classical features achieved moderate to strong predictive performance, with AUROC values ranging from 0.813 to 0.875 on the Validation dataset and 0.817 to 0.850 on the isolated independent Test dataset. The AUROC values ranged from 0.836 to 0.882 during 10-fold cross validation on the Training dataset. While these features provide interpretable descriptors based on physicochemical properties, their discriminative capacity was notably lower than the deep embeddings. Within this category, the Fusion Model (FM) performed marginally better than any other model, while the non-linear models like Random Forest (RF) and Support Vector Machine (SVM) outperformed the Logistic Regression (LR) baseline (e.g., Classical-SVM achieving a Test AUROC of 0.827 compared to Classical-LR’s 0.817). This trend empirically demonstrates that while classical features capture relevant biological signals, these signals are wrapped in a highly multi-collinear and non-linear geometry due to the massive variance dispersion detailed in Section 3.1.1, thereby requiring non-linear classifiers with localized mapping functions to resolve their convoluted boundaries.

##### 3.2.1.2 Impact of deep ESM embeddings

The transition to deep ESM embeddings resulted in a substantial and consistent performance uplift across all evaluated models. Validation AUROC values exceeded 0.90 for all the architectures, with the NN model achieving the highest performance at 0.971, marginally better than the Fusion Model (FM). In 10-fold cross validation, the Fusion Model (FM) performed the best with an AUROC value of 0.968. This exceptional performance carried over to the independent Test dataset, where Deep-NN and Deep-FM secured near-optimal test AUROCs of 0.963 and 0.966, respectively. Unlike the classical features, which often showed a disparity between precision and recall (e.g., Classical-SVM reached 0.864 recall but only 0.718 precision on the Test set), the deep embeddings provided a more balanced performance profile. For instance, the Neural Network (NN) using deep embeddings achieved a recall of 0.909 alongside a much higher precision of 0.930 on the Validation split, maintaining a highly robust 0.930 recall and 0.893 precision on the hidden Test pool.

##### 3.2.1.3 Linear separability

Notably, the Logistic Regression (LR) model saw its MCC increase from 0.501 (classical features) to 0.774 (deep embeddings) on the Validation dataset, and from 0.505 to 0.727 on the Test dataset. Likewise, the Fusion Model (FM) gained significantly in MCC from 0.612 (classical features) to 0.846 (deep embeddings) on validation data. These significant gains indicate that the pretrained protein language model (ESM) effectively projects sequence information into a high-dimensional space where AMR-related signals are more readily accessible, even to simple linear classifiers. Crucially, this net increase of +0.118 Test AUROC units when migrating from Classical-LR (0.817) to Deep-LR (0.935) provides the definitive mathematical proof that deep transformer layers unwrap complex sequence dependencies. It clarifies that while raw unscaled variance trends might create an illusion of single-axis dominance, the actual class boundaries become linearly separable only within the latent coordinate system optimized by the language model, allowing simple linear hyperplanes to directly compete with complex non-linear architectures.

Overall, the comparative analysis in Supplementary Tables S2, S3 and Figure 2 demonstrates that deep embeddings provide a more comprehensive and robust representation of the protein sequence space for AMR prediction, significantly surpassing the baseline established by traditional physicochemical and compositional descriptors.

#### 3.2.2 Overall model performance on the validation dataset and 10-fold cross validation

The evaluation of classical and deep representations revealed that all models achieved strong predictive performance, substantially exceeding random-label baselines and confirming the presence of meaningful biological signals within the individual and integrated feature space. A critical observation from our comparative analysis is that deep ESM embedding features consistently outperformed classical sequence derived features across all model architectures, reinforcing the superiority of deep representations for alignment-free AMR signature detection.

The NN model utilizing deep ESM embeddings emerged as the top-performing configuration, achieving a validation AUROC of 0.971 and an MCC of 0.842, as depicted in Figure 2 and summarized in Supplementary Table S2. While lower than deep embeddings, classical features trained on RF or SVM still provided strong discriminative power (AUROC > 0.850), suggesting that traditional physicochemical descriptors retain relevant, albeit less comprehensive, signal. Combining classical and deep representations yielded highly stable results; for example, the RF model reached its peak validation recall of 0.956 using the integrated set, indicating a high sensitivity for identifying resistance-associated proteins (Figure 2 and Supplementary Table S2). The 10-fold cross validation analysis on Training dataset (*n* = 4,269) demonstrated better stability when compared to the independent validation dataset (*n* = 909). For the NN model, the AUROC remained virtually identical between the 10-fold cross validation (0.965) and the Validation dataset (0.971). This consistency across different data partitions suggests that the alignment-free workflow successfully captures generalizable patterns rather than over-optimizing for specific subsets.

In this study, the Validation dataset was used exclusively for intermediate model evaluation and hyperparameter optimization. By identifying the best-performing set of hyperparameters through these evaluation metrics, we ensured that the final assessment on the held-out Test dataset provides an unbiased estimate of the system’s ability to detect AMR signatures from previously unseen protein sequences.

#### 3.2.3 Robustness and overfitting assessment

Multiple lines of evidence indicate that the reported performance reflects genuine biological signals rather than overfitting or data leakage. First, mandatory sanity checks using randomized class labels and scrambled sequences, confirming the absence of spurious predictive bias. First, to eliminate the possibility that the reported accuracies were driven by pseudo-random initialization anomalies or data partitioning splits, a multi-seed sensitivity analysis was executed across 10 completely independent pipeline iterations. As detailed in Supplementary Table S4, the resulting metric distributions show vanishingly narrow error bounds across all evaluated representations. For instance, our kernel baseline utilizing pure deep embeddings (Deep-SVM) achieved an exceptionally stable performance across distinct seeds, demonstrating a Validation AUROC of 0.9787 ± 0.0000 and a final hidden Independent Test AUROC of 0.9655 ± 0.0000, while the Deep-NN architecture converged at a Test AUROC of 0.9625 ± 0.0028. This extreme consistency empirically proves that the predictive superiority of the transformer-derived embeddings is an invariant property of the optimized sequence space.

Second, all models were trained exclusively on the same Training dataset, with the Validation dataset withheld for feature scaling, model fitting, and hyperparameter optimization before final verification on the locked Test dataset. In addition, the strong agreement between evaluation metrics across models suggests balanced performance rather than optimization toward a single metric. The absence of extreme discrepancies between training loss and validation performance further supports model generalizability.

Finally, to verify the complete absence of spurious predictive biases or data leakage artifacts, we conducted strict two-level negative control sanity checks. Decoupling the feature space from the classification target via a randomized label permutation caused classification performance to collapse into absolute random guessing, yielding a true baseline null Validation AUROC of 0.5240. Similarly, passing sequences through a multi-tier window-shuffling algorithm, disrupting 10% of residue locations at the monopeptide, dipeptide, and tripeptide block levels while keeping global sequence lengths and bulk amino acid counts perfectly unchanged, resulted in a complete drop in predictive power across all six learning architectures, with performance falling close to an AUROC of 0.50. Because the models instantly fail when localized biological grammar and linguistic contexts are broken, this comprehensive assessment confirms that our workflow is learning native, structurally generalizable antimicrobial resistance signatures rather than exploiting background dataset noise.

### 3.3 Performance evaluation of trained models on test dataset

To assess the generalization ability of the trained models, final performance was evaluated on a strictly held-out Test Dataset (*n* = 908, 451 AMR-positive and 457 AMR-negative) that was not used at any stage of model training, feature scaling, or hyperparameter selection. This evaluation provides an unbiased estimate of real-world predictive performance.

#### 3.3.1 Generalization performance on the held-out test dataset

The evaluation on the strictly held-out Test Dataset confirmed that all models demonstrated robust and consistent generalization to unseen sequences (Supplementary Table S3). The results further validate the efficacy of alignment-free strategies, with deep embedding representations significantly outperforming classical features in capturing AMR signals.

The Fusion Models across all the feature spaces performed better than the individual models. The Deep-FM (Fusion Model utilizing deep embeddings) achieved the highest overall performance almost across all metrics, marginally better than Deep-NN, with an AUROC of 0.966, an MCC of 0.818, and an F1-score of 0.911. This underscores the superior capability of deep-learning-based embeddings to extract discriminative features for AMR prediction. While the Deep-RF model showed exceptional sensitivity (Recall of 0.927), the Integrated-RF configuration achieved the highest overall recall of 0.945. This suggests that while deep embeddings drive high precision and AUROC, the addition of classical features may slightly bolster sensitivity in specific ensemble-based architectures. The Deep-LR model maintained highly competitive performance (AUROC of 0.935, MCC of 0.727). The success of this linear baseline indicates that deep representations effectively map protein sequences into a feature space where resistance signals are largely linearly separable, facilitating efficient classification. Models relying solely on classical sequence-derived features (e.g., Classical-SVM and Classical-RF) provided a solid performance baseline (AUROC > 0.82), yet they lagged deep embedding representations by a significant margin.

Consistent with our validation findings, the performance metrics on the held-out Test dataset closely mirror those observed during the optimization phase, as summarized in Supplementary Table S3. This high degree of stability across different data partitions confirms that the alignment-free workflow, particularly when leveraging deep embeddings, provides a reliable and unbiased estimate for AMR detection from novel protein sequences. To definitively eliminate potential dataset partitioning bias and confirm that these out-of-sample performance ceilings represent structurally stable characteristics of the descriptor space rather than single-split anomalies, a comprehensive Monte Carlo resampling validation was executed across 50 independent randomized shuffles (Supplementary Table S5). For each randomized iteration, the entire sequence database was dynamically re-partitioned into a fresh 70:15:15 training, threshold validation, and testing split, executing localized parameter fitting strictly within the training loops and collecting true out-of-sample metrics exclusively from the untouched test segments. The aggregated metrics across all 50 iterations demonstrate remarkable performance stability and minimal dispersion across all evaluated machine learning architectures. The Random Forest model achieved peak predictive stability, yielding a mean accuracy of 0.887 (± 0.0112) and a Matthews Correlation Coefficient (MCC) of 0.777 (± 0.0214), closely followed by the Support Vector Machine (0.867 ± 0.0127 accuracy; 0.736 ± 0.0248 MCC) and the feed-forward Neural Network (0.858 ± 0.0130 accuracy; 0.719 ± 0.0248 MCC). The linear baseline (Logistic Regression) remained highly competitive, maintaining a mean accuracy of 0.848 (± 0.0116) and a tight MCC standard deviation band of ± 0.0224. This consistently narrow variance across 50 completely unique random allocations provides rigorous, empirical proof that the underlying alignment-free signatures are highly resilient to random initialization and split variations, successfully reinforcing the structural robustness of the final validation suite.

#### 3.3.2 Feature integration and multi-space representation dynamics

To evaluate whether combining distinct representational spaces creates a synergistic effect, we systematically analyzed the predictive performance of the Integrated Feature space (combining 8,435 classical descriptors with 1,280 deep embeddings). Empirically, the integrated feature set consistently achieved strong results, outperforming the baseline classical features across all classifiers (e.g., Integrated-SVM achieving a Test AUROC of 0.904 compared to Classical-SVM’s 0.827). However, a key finding of this architectural evaluation is that the Only Deep ESM Embedding models alone consistently and systematically outperformed the larger Integrated Feature sets across all learning paradigms. While initially counter-intuitive, a deep geometric and statistical examination of the features explains why integration leads to performance degradation. This behavior is driven by two main factors:

1. Geometric scale mismatches and scalar swamping: deep ESM-2 embeddings are highly optimized, mathematically unified vectors bounded within a tight, relatively numerical spectrum. In contrast, the classical descriptors encompass raw scales (with class-wise mean differences for mol_weight spanning 9,334.07 units and seq_len spanning 84.07 units on average). When concatenated into an integrated representation, the massive unscaled variance of these raw physical metrics introduces a dominant source of background noise. This numeric noise essentially ‘swamps’ the delicate, localized semantic coordinates captured by the language model, distorting the classification hyper-plane.
2. Multi-collinearity and feature dilution: as mapped out via our Standardized Volcano Plot (Supplementary Figure S2) using Cohen’s d effect sizes, while both individual feature sets carry true biological signals, they operate under completely different dimensional paradigms. Standardized PCA metrics confirm that the classical space is highly diffused and multi-collinear, scattering its variance across 3,436 components to reach the 95% milestone. Because the Integrated space directly inherits this decentralized structure, it expands the required dimensionality to 3,432 components, severely diluting the clean, compact 362-component representation of the pure embeddings.

To further investigate the localized feature-level interactions driving this phenomenon, we compiled class-wise statistics and mapped the statistical significance (−log_10_ Adjusted *p*-value) directly against the Standardized Effect Size (Cohen’s *d*) in Supplementary Figure S2. Utilizing this uniform, standardized metric isolates two vital architectural insights regarding feature integration:

Signal density of deep embeddings: the deep ESM-2 embedding coordinates exhibit remarkable statistical significance, with adjusted *p*-values scaling intensely up to the 10^−120^ range across a tightly balanced effect spectrum. This structural concentration confirms that the transformer language model architecture captures exceptionally clean, dense, and localized evolutionary drivers of resistance without spreading noise.
Concatenation dilemma: while traditional classical biochemical features (such as sequence length, molecular weight, and instability index) also establish high individual statistical significance, their underlying features operate on completely divergent, unscaled geometric axes. When directly appended into the combined ‘Integrated’ space without complex mathematical mapping layers, these unscaled physical distributions introduce severe multi-collinearity and global variance distortions. This background data noise effectively swamps the high-precision latent vectors generated by the language model layers.

Rather than enriching the classifier, direct concatenation introduces structural redundancy, multi-collinearity, and scale distortions. This scatters the decision boundaries across thousands of unnecessary dimensions, forcing the classification models to expend optimization resources on resolving collinear noise. Consequently, the pure, pre-optimized deep embedding feature space maintains a cleaner and superior predictive accuracy on its own.

#### 3.3.3 Error analysis and model behavior

**Figure 3:**
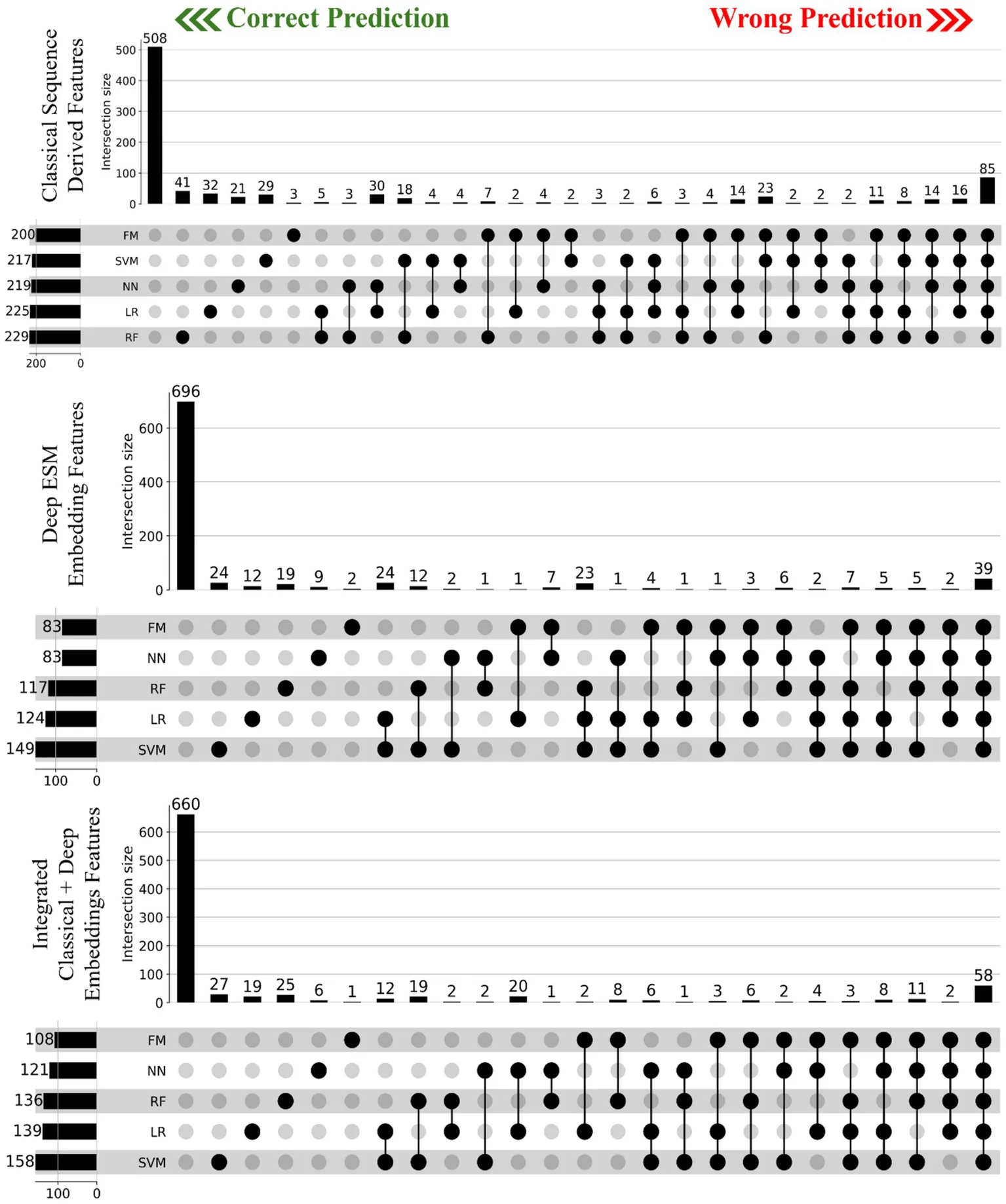
An UpSet plot intersection analysis of model classification performance on the independent test dataset (*n* = 908). This visualization maps the shared patterns of correct and incorrect predictions across the four primary models, viz. Logistic Regression (LR), Support Vector Machine (SVM), Random Forest (RF), and Neural Network (NN) alongside the stacked Fusion Model (FM). Vertical bars indicate the number of proteins falling into each prediction category. Connected nodes in the lower panels define the specific model combinations tracking performance. Gray circles denote correct classifications, while black circles denote incorrect predictions. Column groups on the far-left side of the matrix (where all circles are gray) indicate consensus successes, where all evaluated methods correctly identified the target protein. Conversely, column groups on the far-right side of the matrix (where all circles are black) highlight collective failures, where all predictive models failed simultaneously to classify the sequence.

A comparative evaluation of persistent misclassifications across classical sequence-derived features (*n* = 85), deep ESM embeddings (*n* = 39), and integrated representations (*n* = 58) revealed 17 instances where the biochemical signal remained unresolved across all feature spaces (Figure 3). This cohort comprised 13 AMR-positive false negatives and 4 AMR-negative false positives. To investigate the molecular basis of these discrepancies, we performed phylogenetic analysis and extracted high-resolution structural metrics using a standalone implementation of AlphaFold3.

**Figure 4:**
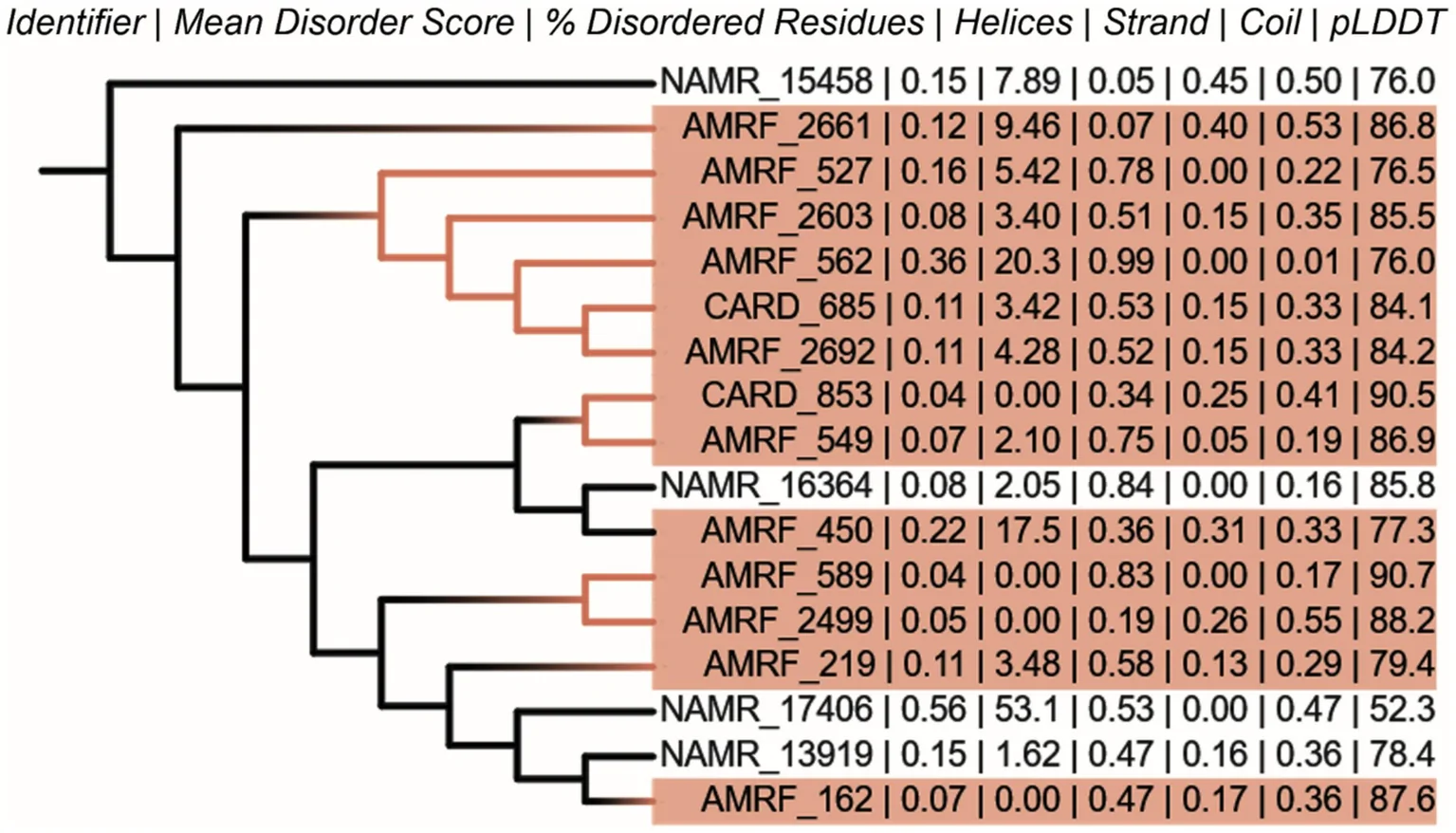
Maximum-likelihood phylogenetic tree and integrated biophysical profile of persistently misclassified AMR candidates. The evolutionary tree traces the divergence constraints of the 17 sequences that remained unresolved across all evaluated numerical feature spaces. Red-highlighted branches track AMR-positive false negatives. Leaf nodes are labeled dynamically using a standardized, easy-to-scan data string mapping structural properties calculated from the structural models by AlphaFold3. Highly ordered false negatives (e.g., sister-clade AMRF_2692 and CARD_685, sharing a branch length of 0.12011) exhibit clear alpha-helical dominance (helices ≥ 0.51) and low disorder, exposing instances where resistant mechanisms structurally mimic non-resistant housekeeping protein geometries. Conversely, false-positive outgroups or nested nodes reveal structural disorder as a driver of predictive error. Candidate NAMR_15458 forms the absolute outgroup (branch length = 1.07576) with an atypical high-strand fraction (0.45), while the highly disordered NAMR_17406 (53.08% sequence disorder, 52.32 pLDDT) clusters deeply inside a core AMR-positive clade, showing how regional conformational flexibility creates chaotic contextual pathways that generate false resistance signals. The complete raw metric parameters are compiled in Supplementary Table S6.

The phylogenetic topologies and integrated biophysical properties of these 17 proteins are illustrated in Figure 4, where individual leaf nodes are explicitly annotated via an integrated data vector detailing sequence identifiers, mean disorder scores, percent disordered residues, secondary structure fractions (helices, strands, coils), and model prediction confidence (pLDDT). The full raw matrix tracking these parameters is compiled in Supplementary Table S6.

Phylogenetic reconstruction reveals that the misclassified sequences are scattered across distinct clades, where they are often paired with non-resistant structural mimics. For instance, the highly ordered AMR-positive false negatives AMRF_2692 and CARD_685 form a tightly conserved sister-clade sharing a branch length of just 0.12011; both display highly similar node labels with helical fractions exceeding 0.51 and low internal disorder scores under 0.11, confirming their close structural alignment.

Conversely, the AMR-negative false positive cohort is evolutionarily split across the tree, exposing multiple ways that structural noise can mislead numerical representations. As displayed on the bottom outgroup branch (branch length = 1.07576), candidate NAMR_15458 diverges heavily from the helical dominance of the core resistance cohort, showing an exceptionally low helical fraction of 0.05 and a high strand fraction of 0.45 at a pLDDT confidence of 76.0. In sharp contrast, the most severely unstable false positive, NAMR_17406, is phylogenetically nested deep within a core AMR-positive clade, branching tightly with AMRF_162 and NAMR_13919. As detailed on its integrated node label, NAMR_17406 exhibits a high mean disorder score of 0.5572 with 53.08% of its sequence flagged as unstructured, causing its structural prediction confidence (pLDDT) to drop to a weak 52.32. This close evolutionary clustering indicates that its high regional flexibility creates chaotic contextual paths that mimic genuine resistance vectors, misleading the numerical feature spaces into generating false resistance signals despite its lack of a functional resistance phenotype.

Overall, this multi-layered error analysis demonstrates that the predictive models prioritize biologically relevant macromolecular signatures over trivial sequence artifacts. The high degree of consensus among divergent architectures suggests a robust, learned representation of AMR-associated functional characteristics. However, the identification of a recalcitrant subset (*n* = 17) characterized by localized multi-collinear mimicry or high-fraction intrinsically disordered regions (IDRs), as confirmed by AlphaFold3 modeling and phylogenetic clustering, highlights the current boundaries of numerical feature spaces. The existence of model-specific sensitivities toward these unique protein folds justifies a tiered, consensus-based prioritization strategy. Such an approach, consensus-of-three provides a more comprehensive framework for resolving the sequence-structure–function relationships of highly divergent or novel resistant macromolecules, ensuring higher confidence in candidates selected for experimental validation.

### 3.4 Benchmarking with existing AMR prediction approaches

#### 3.4.1 Rationale and selection of benchmarking methods

To contextualize the classical and deep embedding features evaluation, we benchmarked it against diverse homology-, machine learning- and deep learning-based AMR detection tools. The objective was to position this study within the landscape of alignment-free approaches that generalize beyond sequence homology. Selection criteria included the use of learned representations, applicability to protein sequences, and pipeline availability. We selected DeepARG (implements neural networks classifier along with alignments to the known AMR proteins) (Arango-Argoty et al., 2018), PLM-ARG (implements pretrained protein language model embeddings) (Wu et al., 2023), and ProtAlign-ARG (implements pretrained protein language model embeddings along with alignment features) (Ahmed et al., 2025) as representative data-driven methods. Additionally, AMRFinder+ (Feldgarden et al., 2021) was included to provide a baseline for a purely homology-based approach, despite its reliance on the same underlying database used for our dataset compilation.

Furthermore, we acknowledge that the performance of certain benchmarking tools may be positively influenced by potential overlaps between their original training sets and our test dataset. This focus on AI/ML-driven methods ensures a methodologically consistent assessment of generalization capacity for identifying novel or divergent resistance proteins.

#### 3.4.2 Comparative performance on the held-out test dataset

Benchmarking was performed on a balanced held-out Test Dataset comprising 908 proteins (451 AMR-positive and 457 AMR-negative). The primary finding of this evaluation is that models leveraging deep embeddings consistently outperformed traditional homology-based and hybrid benchmarked tools across key performance dimensions. Notably, the Deep-NN configuration achieved the highest overall metrics in this study (AUROC: 0.963, MCC: 0.818, F1: 0.911), demonstrating that deep representations capture discriminative signals more effectively than current specialized AMR prediction frameworks. Our evaluation highlights significant performance trade-offs in existing tools compared to the evaluated representations.

Established tools like AMRFinderPlus, PLM-ARG, and DeepARG exhibited extremely high precision (up to 1.000) but suffered from limited sensitivity (Recall ranging from 0.443 to 0.510). In contrast, models using deep and integrated representations achieved a more robust balance; for instance, Deep-RF maintained a high recall of 0.927 while keeping an AUROC of 0.942. While ProtAlign-ARG achieved relatively high sensitivity (0.789), it was hampered by a high false-positive rate, resulting in the lowest precision (0.531) and AUROC (0.554) among the benchmarks. Conversely, even our Classical-NN model (using only traditional features) achieved superior discriminative power (AUROC: 0.840) compared to the specialized ProtAlign-ARG framework.

The high performance across all models trained on deep embeddings (AUROC ≥ 0.89) versus the lower performance of classical features (AUROC range: 0.81–0.84) confirms that the gains are driven primarily by the feature representation strategy rather than model-specific optimizations. As summarized in Table 4 and Supplementary Table S3, deep embeddings, whether used alone or integrated with classical features, provide a significantly more balanced discrimination between AMR and non-AMR proteins. These results suggest that alignment-free deep representations can overcome the conservative nature of homology-driven methods without sacrificing the specificity required for reliable AMR annotation.

**Table 4:** Comparative performance of AMR prediction methods on the held-out test dataset, in terms of sensitivity, specificity, AUROC, precision, recall, F1-score, and MCC metrics.

Method	Strategy	AUROC	MCC	F1	Precision	Recall
Classical-FM	alignment-free, classical features	0.850	0.568	0.799	0.738	0.871
Deep-FM	alignment-free, deep embeddings	**0.966**	**0.818**	**0.911**	**0.891**	**0.932**
Integrated-FM	alignment-free, integrated features	0.943	0.765	0.887	0.853	0.923
AMRFinder+	rule based homology-driven framework	0.752	0.588	0.671	1.000	0.505
DeepARG	alignment + hybrid neural network	0.719	0.719	0.613	0.990	0.443
PLM-ARG	pretrained PLM embeddings	0.753	0.580	0.673	0.991	0.510
ProtAlign-ARG	PLM embeddings + alignment features	0.554	0.554	0.635	0.531	0.789

For the benchmark methods, the AUROC was calculated based on single point approximation by considering the mean value of sensitivity and specificity. Deep embedding based fusion model perfomed the best and its evaluation metrics are highlighted in bold.

### 3.5 Performance evaluation on external dataset

To verify the real-world deployability of the framework, the pre-trained, locked machine learning models were evaluated on external dataset (*n* = 527 sequences). The comparative performance metrics mapping classification generalizability across the Classical, Deep Embedding, and Integrated feature spaces are summarized in Supplementary Table S7.

The resulting classification distributions confirm the generalizability of the alignment-free pipeline across independent data repositories. Within the standalone deep embedding space, the Deep-NN architecture achieved a strong performance profile with an AUROC of 0.9717 and an MCC of 0.7883, while maintaining a balanced precision of 0.9104 and recall of 0.9408. This stable performance indicates that the pretrained transformer model successfully preserves contextual sequence properties across distinct protein divisions. Furthermore, consistent with our previous architectural findings, the Integrated feature space showed lower overall accuracy compared to pure embeddings across matching model types, confirming that direct concatenation scales up multi-collinear and scalar distortions.

### 3.6 Structural prioritization and strategy for experimental validation

The practical utility of any computational framework for antimicrobial resistance (AMR) lies in its capacity to guide downstream experimental characterization of resistant macromolecules. Based on our intersectional error analysis (Section 3.4.2) and benchmarking against homology-based tools, we propose a multi-modal consensus strategy for prioritizing protein candidates for biochemical and functional validation. This framework balances the identification of high-confidence resistance determinants with the discovery of novel, divergent mechanisms that elude traditional sequence alignment.

#### 3.6.1 High-confidence functional annotation

Protein candidates classified as AMR-positive by multiple model architectures that also exhibit significant hits in similarity-based tools (e.g., AMRFinderPlus) should be prioritized for standard functional validation. Our UpSet plot analysis revealed a high degree of consensus (680/908 instances) across diverse machine learning frameworks, signifying a robust and conserved biochemical signal (Figure 3). When independent methodologies, specifically alignment-based homology and embedding-based contextual representations, converge on a single macromolecule, the probability of a true positive functional assignment is maximized. These candidates typically represent established protein families with well-characterized resistance-relevant motifs.

#### 3.6.2 Consensus-based discovery of divergent macromolecules

For large-scale proteomic screening, we propose a “Consensus-of-Three” rule (requiring agreement among at least three of the five models). This approach optimizes the balance between sensitivity and the high costs associated with experimental protein characterization. Implementing this rule expanded the detection accuracy to 89% (809/980 instances) for deep embedding-based representations.

Beyond raw predictive performance, this consensus framework provides a structured biologically informed strategy for interpreting model outputs. By integrating heterogeneous feature spaces, researchers can navigate the trade-offs between sensitivity and specificity, facilitating the identification of novel resistance macromolecules that lack close homologs in existing databases. This pipeline effectively transitions AMR research from a sequence-dependent paradigm to one focused on the functional and structural properties of the protein.

### 3.7 Limitations and future directions

Despite the robust performance of the deep embedding-based alignment-free framework, certain limitations define its current scope and guide future methodological extensions. The present study focuses exclusively on protein sequence-level modeling. While these features capture substantial functional information, resistance phenotypes are often modulated by the broader biochemical environment, including molecular stoichiometry, regulatory interactions, and post-translational modifications. The absence of such higher-order context may limit resolution where resistance arises from expression dynamics rather than primary structural variation. Furthermore, while feature concatenation proved effective, advanced multimodal fusion strategies, such as attention-based mechanisms, could better resolve fine-grained interactions between physicochemical descriptors and the latent spaces of protein language models. Specifically, simple early concatenation forces downstream classification layers to resolve multi-collinear and scalar disparities independently, which can introduce artifactual noise. A natural architectural improvement is the transition to an Attention-Gated Multi-Modal Fusion Network. In this proposed framework, the 8,435-dimensional classical descriptor vector and the 1,280-dimensional ESM-2 embedding vector would pass through independent neural network mapping projections to equalize their dimensional ranks. These mapped spaces would then feed into a cross-attention module that calculates a localized alignment matrix, learning the exact interaction weights between raw biochemical properties and global semantic contexts before classification. While our current stacking meta-classifier (Fusion Model) serves as an effective intermediate learned baseline utilizing the predictions of LR, SVM, RF, and NN architectures, moving toward deep attention-guided gating will be necessary to exploit cross-space synergies fully. Additionally, this study employs binary classification and does not yet stratify results by specific macromolecular mechanisms, such as enzymatic degradation, efflux pump assembly, or target site modification. This binary scope fundamentally restricts the framework from resolving downstream phenotypic characteristics and drug-class specific interactions. Transitioning to full mechanism-level stratification presents distinct data-driven and algorithmic challenges, primarily driven by severe class imbalance and data sparsity across rarer resistance mechanisms, alongside the biological reality of multi-functional proteins that act through overlapping defensive pathways simultaneously. Standard flat multi-class models fail to capture these intricate, non-mutually exclusive relationships, as forcing a sequence into a single mechanism category introduces artificial optimization constraints into the latent representation space.

Future research will address these limitations through several natural extensions. (i) Transitioning from binary to multi-class frameworks to predict specific biochemical resistance mechanisms and antibiotic classes, thereby enhancing molecular relevance. (ii) Incorporating 3D structural information and folding dynamics, potentially via AlphaFold3 integration, to enrich the representation of functional binding pockets. (iii) Systematically evaluating performance across diverse bacterial taxa to identify genus-specific variations in macromolecular architecture. Collectively, these directions position this evaluation as a modular foundation for future alignment-free systems that move toward mechanistically informed, structure-aware resistance modeling of biological macromolecules.

## 4 Discussion and conclusion

The present study demonstrates that antimicrobial resistance (AMR) signatures can be reliably resolved from protein sequences using an alignment-free framework centered on macromolecular feature representations. By utilizing both classical sequence-derived descriptors and deep protein language model (pLM) embeddings, we effectively captured the physicochemical and contextual determinants that define resistant proteins. The robust generalization observed across inter-partition divergence (~37–44% identity) confirms that the models are identifying inherent biochemical patterns rather than relying on homologous memorization.

Analysis of the feature spaces revealed the complementary nature of these protein representations. While classical descriptors provide discrete summaries of amino acid composition and residue-level properties, they possess limited capacity to encode long-range interactions. In contrast, deep pLM embeddings capture high-dimensional contextual information related to protein folding and functional domains. The observed collapse in effective dimensionality upon feature integration suggests that the dominant biological variance, the signatures of resistance, is most efficiently captured when these modalities are combined. Although deep embeddings served as the primary drivers of predictive power, the inclusion of classical features stabilized the decision boundaries, likely by providing a biochemical baseline that grounds the abstract latent space of the pLM.

The consistency of model performance on independent datasets, reinforced by randomized-label and scrambled-sequence controls, confirms that the identified signatures are rooted in the molecular architecture of the proteins. Our error analysis suggests that misclassifications often reflect biological ambiguity, such as proteins whose resistance phenotypes are modulated by the broader genomic context or expression levels, rather than technical artifacts. Furthermore, while alignment-based tools showed high precision, their reduced sensitivity toward divergent sequences highlights the necessity of representation-learning approaches that can look beyond primary sequence homology to the underlying functional signatures of the macromolecule.

In conclusion, this study provides a systematic evaluation of alignment-free protein representations for the detection of AMR-associated functional signatures. Our findings establish that while deep embeddings capture the majority of the resistance-relevant signal, the integration of classical biochemical descriptors enhances the robustness and stability of the classification. This synergy underscores the importance of utilizing heterogeneous feature spaces to probe the complex sequence-structure–function relationships in biological macromolecules. These results provide a principled foundation for future research incorporating high-resolution structural data and mechanistic stratification to further refine biologically informed pipelines for the discovery of novel resistant macromolecules.

## Data Availability

The datasets presented in this study can be found in online repositories. The names of the repository/repositories and accession number(s) can be found in the article/Supplementary material.

## References

[ref1] Abramson J. Adler J. Dunger J. Evans R. Green T. Pritzel A. . (2024). Accurate structure prediction of biomolecular interactions with AlphaFold 3. Nature 630, 493–500. doi: 10.1038/s41586-024-07487-w, 38718835 PMC11168924

[ref2] Ahmed S. Emon M. I. Moumi N. A. Huang L. Zhou D. Vikesland P. . (2025). ProtAlign-ARG: antibiotic resistance gene characterization integrating protein language models and alignment-based scoring. Sci. Rep. 15:30174. doi: 10.1038/s41598-025-14545-4, 40825968 PMC12361412

[ref3] Alcock B. P. Huynh W. Chalil R. Smith K. W. Raphenya A. R. Wlodarski M. A. . (2023). CARD 2023: expanded curation, support for machine learning, and resistome prediction at the comprehensive antibiotic resistance database. Nucleic Acids Res. 51, D690–D699. doi: 10.1093/nar/gkac920, 36263822 PMC9825576

[ref4] Alem K. Dagnew M. Gizachew M. Gelaw B. Moges F. (2025). Environmental antimicrobial resistance: key drivers, hotspots, innovative strategies, and challenges in the fight against superbugs. MicrobiologyOpen 14:e70067. doi: 10.1002/mbo3.70067, 41085275 PMC12519517

[ref5] Arango-Argoty G. Garner E. Pruden A. Heath L. S. Vikesland P. Zhang L. (2018). DeepARG: a deep learning approach for predicting antibiotic resistance genes from metagenomic data. Microbiome 6:23. doi: 10.1186/s40168-018-0401-z, 29391044 PMC5796597

[ref6] Bhattacharya S. Padhi A. K. Junghare V. Das N. Ghosh D. Roy P. . (2021). Understanding the molecular interactions of inhibitors against Bla1 beta-lactamase towards unraveling the mechanism of antimicrobial resistance. Int. J. Biol. Macromol. 177, 337–350. doi: 10.1016/j.ijbiomac.2021.02.069, 33582216

[ref7] Bilal H. Khan M. N. Khan S. Shafiq M. Fang W. Khan R. U. . (2025). The role of artificial intelligence and machine learning in predicting and combating antimicrobial resistance. Comput. Struct. Biotechnol. J. 27, 423–439. doi: 10.1016/j.csbj.2025.01.006, 39906157 PMC11791014

[ref8] Darby E. M. Trampari E. Siasat P. Gaya M. S. Alav I. Webber M. A. . (2023). Molecular mechanisms of antibiotic resistance revisited. Nat. Rev. Microbiol. 21, 280–295. doi: 10.1038/s41579-022-00820-y, 36411397

[ref9] Eisenreich W. Rudel T. Heesemann J. Goebel W. (2022). Link between antibiotic persistence and antibiotic resistance in bacterial pathogens. Front. Cell. Infect. Microbiol. 12:900848. doi: 10.3389/fcimb.2022.900848, 35928205 PMC9343593

[ref10] Feldgarden M. Brover V. Fedorov B. Haft D. H. Prasad A. B. Klimke W. (2022). Curation of the AMRFinderPlus databases: applications, functionality and impact. Microb. Genom. 8:832. doi: 10.1099/mgen.0.000832, 35675101 PMC9455714

[ref11] Feldgarden M. Brover V. Gonzalez-Escalona N. Frye J. G. Haendiges J. Haft D. H. . (2021). AMRFinderPlus and the reference gene catalog facilitate examination of the genomic links among antimicrobial resistance, stress response, and virulence. Sci. Rep. 11:12728. doi: 10.1038/s41598-021-91456-0, 34135355 PMC8208984

[ref12] Frazer J. Notin P. Dias M. Gomez A. Min J. K. Brock K. . (2021). Disease variant prediction with deep generative models of evolutionary data. Nature 599, 91–95. doi: 10.1038/s41586-021-04043-8, 34707284

[ref13] Goldfarb T. Kodali V. K. Pujar S. Brover V. Robbertse B. Farrell C. M. . (2025). NCBI RefSeq: reference sequence standards through 25 years of curation and annotation. Nucleic Acids Res. 53, D243–D257. doi: 10.1093/nar/gkae1038, 39526381 PMC11701664

[ref14] Gschwind R. Ugarcina Perovic S. Weiss M. Petitjean M. Lao J. Coelho L. P. . (2023). ResFinderFG v2.0: a database of antibiotic resistance genes obtained by functional metagenomics. Nucleic Acids Res. 51, W493–W500. doi: 10.1093/nar/gkad384, 37207327 PMC10320180

[ref15] Hayes T. Rao R. Akin H. Sofroniew N. J. Oktay D. Lin Z. . (2025). Simulating 500 million years of evolution with a language model. Science 387, 850–858. doi: 10.1126/science.ads0018, 39818825

[ref16] Huang Y. Niu B. Gao Y. Fu L. Li W. (2010). CD-HIT suite: a web server for clustering and comparing biological sequences. Bioinformatics 26, 680–682. doi: 10.1093/bioinformatics/btq003, 20053844 PMC2828112

[ref17] Jean S.-S. Harnod D. Hsueh P.-R. (2022). Global threat of Carbapenem-resistant gram-negative Bacteria. Front. Cell. Infect. Microbiol. 12:823684. doi: 10.3389/fcimb.2022.823684, 35372099 PMC8965008

[ref18] Kalita P. Lyngdoh D. L. Padhi A. K. Shukla H. Tripathi T. (2019). Development of multi-epitope driven subunit vaccine against *Fasciola gigantica* using immunoinformatics approach. Int. J. Biol. Macromol. 138, 224–233. doi: 10.1016/j.ijbiomac.2019.07.024, 31279880

[ref19] Kant R. Kumar N. Malik Y. S. Everett D. Saluja D. Launey T. . (2024). Critical insights from recent outbreaks of *Mycoplasma pneumoniae*: decoding the challenges and effective interventions strategies. Int. J. Infect. Dis. 147:107200. doi: 10.1016/j.ijid.2024.107200, 39117175

[ref20] Kaushik R. Kant R. Christodoulides M. (2023). Artificial intelligence in accelerating vaccine development - current and future perspectives. Front. Bacteriol. 2:1258159. doi: 10.3389/fbrio.2023.1258159

[ref21] Kaushik R. Re S. (2026). Artificial intelligence directed computational protein design: lessons from COVID-19 for pandemic-ready vaccines and antibody therapeutics. J. Pharm. Pharm. Sci. 29:16146. doi: 10.3389/jpps.2026.16146, 42181087 PMC13194078

[ref22] Kaushik R. Zhang K. Y. J. (2020). A protein sequence fitness function for identifying natural and nonnatural proteins. Proteins 88, 1271–1284. doi: 10.1002/prot.25900, 32415863

[ref23] Kaushik R. Zhang K. Y. J. (2022a). ProFitFun: a protein tertiary structure fitness function for quantifying the accuracies of model structures. Bioinformatics 38, 369–376. doi: 10.1093/bioinformatics/btab666, 34542606

[ref24] Kaushik R. Zhang K. Y. J. (2022b). An integrated protein structure fitness scoring approach for identifying native-like model structures. Comput. Struct. Biotechnol. J. 20, 6467–6472. doi: 10.1016/j.csbj.2022.11.032, 36467582 PMC9708444

[ref25] Li X. Mowlaboccus S. Jackson B. Cai C. Coombs G. W. (2024). Antimicrobial resistance among clinically significant bacteria in wildlife: an overlooked one health concern. Int. J. Antimicrob. Agents 64:107251. doi: 10.1016/j.ijantimicag.2024.107251, 38906487

[ref26] Lin Z. Akin H. Rao R. Hie B. Zhu Z. Lu W. . (2023). Evolutionary-scale prediction of atomic-level protein structure with a language model. Science 379, 1123–1130. doi: 10.1126/science.ade2574, 36927031

[ref27] Mall R. Kaushik R. Martinez Z. A. Thomson M. W. Castiglione F. (2025). Benchmarking protein language models for protein crystallization. Sci. Rep. 15:2381. doi: 10.1038/s41598-025-86519-5, 39827171 PMC11743144

[ref28] Mirdita M. Schütze K. Moriwaki Y. Heo L. Ovchinnikov S. Steinegger M. (2022). ColabFold: making protein folding accessible to all. Nat. Methods 19, 679–682. doi: 10.1038/s41592-022-01488-1, 35637307 PMC9184281

[ref29] Muñoz K. A. Ulrich R. J. Vasan A. K. Sinclair M. Wen P.-C. Holmes J. R. . (2024). A gram-negative-selective antibiotic that spares the gut microbiome. Nature 630, 429–436. doi: 10.1038/s41586-024-07502-0, 38811738 PMC12135874

[ref30] Murray C. J. L. Ikuta K. S. Sharara F. Swetschinski L. Robles Aguilar G. Gray A. . (2022). Global burden of bacterial antimicrobial resistance in 2019: a systematic analysis. Lancet 399, 629–655. doi: 10.1016/S0140-6736(21)02724-0, 35065702 PMC8841637

[ref31] Naghavi M. Vollset S. E. Ikuta K. S. Swetschinski L. R. Gray A. P. Wool E. E. . (2024). Global burden of bacterial antimicrobial resistance 1990–2021: a systematic analysis with forecasts to 2050. Lancet 404, 1199–1226. doi: 10.1016/S0140-6736(24)01867-1, 39299261 PMC11718157

[ref32] Santos-Júnior C. D. Torres M. D. T. Duan Y. Rodríguez Del Río Á. Schmidt T. S. B. Chong H. . (2024). Discovery of antimicrobial peptides in the global microbiome with machine learning. Cell 187, 3761–3778.e16. doi: 10.1016/j.cell.2024.05.013 38843834 PMC11666328

[ref33] Shanker V. R. Bruun T. U. J. Hie B. L. Kim P. S. (2024). Unsupervised evolution of protein and antibody complexes with a structure-informed language model. Science 385, 46–53. doi: 10.1126/science.adk8946, 38963838 PMC11616794

[ref34] Smith A. B. Jenior M. L. Keenan O. Hart J. L. Specker J. Abbas A. . (2022). Enterococci enhance Clostridioides difficile pathogenesis. Nature 611, 780–786. doi: 10.1038/s41586-022-05438-x, 36385534 PMC9691601

[ref35] Szymczak P. Zarzecki W. Wang J. Duan Y. Wang J. Coelho L. P. . (2025). AI-driven antimicrobial peptide discovery: mining and generation. Acc. Chem. Res. 58, 1831–1846. doi: 10.1021/acs.accounts.0c00594, 40459283 PMC12177927

[ref36] Tewolde R. Thombre R. Farley C. Nadarajah S. Khan I. Sewell M. . (2025). Comparison of phenotypic and whole-genome sequencing-derived antimicrobial resistance profiles of *Legionella pneumophila* isolated in England and Wales from 2020 to 2023. Antibiotics 14:1053. doi: 10.3390/antibiotics14101053, 41148745 PMC12562041

[ref37] Thombre R. Jangid K. Shukla R. Dutta N. K. (2019). Alternative therapeutics against antimicrobial-resistant pathogens. Front. Microbiol. 10:2173. doi: 10.3389/fmicb.2019.02173 31608028 PMC6761881

[ref38] Wang Z. Koirala B. Hernandez Y. Zimmerman M. Park S. Perlin D. S. . (2022). A naturally inspired antibiotic to target multidrug-resistant pathogens. Nature 601, 606–611. doi: 10.1038/s41586-021-04264-x, 34987225 PMC10321319

[ref39] Wu J. Ouyang J. Qin H. Zhou J. Roberts R. Siam R. . (2023). PLM-ARG: antibiotic resistance gene identification using a pretrained protein language model. Bioinformatics 39:btad690. doi: 10.1093/bioinformatics/btad690, 37995287 PMC10676515

