# Supplementary material for: Computational mapping of resistance-relevant signals in proteins using deep and classical feature spaces
Source: Front Microbiol. 2026 Aug 19;17:1899081. doi: 10.3389/fmicb.2026.1899081 (PMC13534041; doi:10.3389/fmicb.2026.1899081)
Supplement: Supplementary file 2 [file Table_1.DOCX]

**SUPPLEMENTARY NOTE - I**

**Details of Machine Learning Models**

***Logistic Regression:*** Logistic regression was used as a linear baseline classifier to evaluate the separability of antimicrobial resistance (AMR) and non-AMR proteins in the integrated feature space. Logistic Regression was implemented as a linear baseline classifier operating in the high-dimensional feature space derived from sequence-derived descriptors and embedding representations. L2 regularization was applied to control model complexity and prevent overfitting. Regularization strength was fixed at C = 1.0, representing the inverse of the regularization penalty. This value was selected to provide a balanced trade-off between bias and variance without extensive hyperparameter optimization. The saga solver was used due to its scalability in high-dimensional settings and compatibility with large feature matrices. Model convergence was ensured by setting the maximum number of iterations to 5000. Feature standardization (zero mean, unit variance) was applied prior to training to stabilize gradient-based optimization and ensure comparable feature scaling.

***Support Vector Machine:*** Support Vector Machine classifiers were implemented using a radial basis function (RBF) kernel to capture non-linear decision boundaries in the embedding space. The RBF kernel projects input features into a higher-dimensional space where class separation may be more linearly separable. Limited hyperparameter tuning was performed using grid search within the training set under 5-fold cross-validation. The regularization parameter C was evaluated over (0.1, 1, 10), controlling the trade-off between margin maximization and misclassification penalty. The kernel coefficient γ was evaluated over (scale, 0.01, 0.001), determining the influence radius of individual training samples. Probability estimation was enabled to allow computation of AUROC, PR-AUC, Brier Score, and calibration metrics. Feature standardization was applied prior to model fitting due to the distance-based nature of the RBF kernel. SVM provides a strong non-linear baseline while maintaining controlled hyperparameter complexity.

***Random Forest:*** Random Forest models were implemented as an ensemble of 500 decision trees. Each tree was trained on bootstrap samples of the training data, and node splitting was performed using the Gini impurity criterion. At each split, a random subset of features equal to the square root of the total number of features was considered (max_features = sqrt). This strategy reduces inter-tree correlation and improves generalization. Trees were allowed to grow without explicit depth limitation, enabling modeling of complex feature interactions. Random Forest models were trained on unscaled features, as tree-based algorithms are invariant to monotonic transformations of input variables. Random Forest provides a robust ensemble baseline with strong resistance to noise and overfitting in high-dimensional tabular data.

***Neural Network:*** A fully connected feed-forward neural network was implemented using a two-hidden-layer architecture (512 and 256 neurons). Rectified Linear Unit (ReLU) activations were used to promote efficient gradient propagation and mitigate vanishing gradient effects. Optimization was performed using the Adam optimizer, which adaptively adjusts learning rates for individual parameters. L2 regularization (α = 1×10⁻⁴) was applied to reduce overfitting. Early stopping was enabled based on internal validation loss, with 10% of the training data reserved for monitoring convergence. The maximum number of training iterations was set to 500. Feature standardization was applied prior to training to ensure stable optimization dynamics. The neural network provides a non-linear deep learning baseline capable of capturing complex interactions among sequence-derived features.

**SUPPLEMENTARY NOTE – II**

**Overview of Benchmarked Methods**

***AMRFinderPlus:*** It serves as a high-precision, homology-driven framework that identifies resistance genes through a curated database. It operates on explicit decision rules and direct sequence similarity. While this method is considered the "gold standard" for accuracy when dealing with known sequences, it is inherently limited by its reliance on homology; it often struggles to detect divergent proteins or novel resistance mechanisms that do not closely match its existing database.

***DeepARG:*** It utilizes a hybrid neural network approach that bridges traditional alignment with machine learning. It uses the DIAMOND tool to align protein sequences against its proprietary database, converting the resulting bit scores into fixed-length feature vectors for a neural network to process. Although it incorporates deep learning architecture, its predictive power is still fundamentally anchored in alignment-based signals, meaning its effectiveness remains tied to how well a sequence aligns with previously documented genes.

***PLM-ARG:*** It represents a shift toward modern "alignment-reduced" strategies by utilizing pretrained protein language model (PLM) embeddings. Instead of relying solely on direct sequence comparisons, it feeds these high-dimensional embeddings into downstream classifiers. By focusing on the "language" and structural patterns of proteins, this method decreases the dependence on explicit sequence alignment while still benefiting from training on established resistance-labeled datasets.

***ProtAlign-ARG:*** It is a dual-input system that attempts to capture the best of both worlds by combining contextual PLM embeddings with homology-derived alignment features. This integration is designed to maximize recall, ensuring that fewer potential resistance genes are missing. However, this increased sensitivity comes with a trade-off, as the model tends to exhibit a higher rate of false positives compared to more conservative, rule-based systems.

**SUPPLEMENTARY TABLES**

**Table S1.** Table S1. A summary of structural and discriminative properties of classical, deep, and integrated feature spaces. Mean absolute feature correlation was computed across all numeric features within each feature space. Discriminative features were identified using two-sided Mann-Whitney U tests between AMR and non-AMR classes with Benjamini-Hochberg false discovery rate (FDR) correction. The integrated feature space combines classical descriptors and deep ESM embeddings after redundancy reduction and label-safe merging. The PCA (50% / 80% / 95%) columns indicate the number of components required to explain 50%, 80%, and 95% of the total cumulative variance under both raw unscaled and standardized (z-score scaled) data processing conditions.

| Feature Space | PCA Mode | PCA  (50% / 80% / 95%) | Feature Correlation | Features Difference  (FDR < 0.05) | Interpretation |
| --- | --- | --- | --- | --- | --- |
| Classical Features | Unscaled  Scaled | 1 / 1 / 1  750 / 1934 / 3436 | 0.013 |  | Unscaled dominance masked by scale inequalities; scaled view reveals massive collinearity dispersion. |
| Deep Embedding | Unscaled  Scaled | 4 / 42 / 244  18 / 90 / 362 | 0.119 |  | Highly optimized contextual structure; transformer layers compress structural signal into compact orthogonal geometry. |
| Integrated  Features | Unscaled  Scaled | 1 / 1 / 1  658 / 1869 / 3432 | 0.017 | 4,216 | High multi-collinear diffusion; unscaled mix causes variance masking, whereas scaled mix requires non-linear boundaries. |

**Table S2.** Performance of machine learning models evaluated through 10-fold cross validation and independent validation dataset. Validation metrics were used for model selection and hyperparameter tuning. AUROC denotes the area under the receiver operating characteristic curve, MCC denotes Matthew’s correlation coefficient, and F1-score represents the harmonic mean of precision and recall. LR: Logistic Regression, SVM: Support Vector Machine, RF: Random Forest, NN: Neural Network, and FM: Fusion Model. For the reproducibility purpose, the random seed was fixed to 42 for this run.

| Features | Models | 10-Fold Cross Validation (n = 4269) | | | | | Validation Dataset (n = 909) | | | | |
| --- | --- | --- | --- | --- | --- | --- | --- | --- | --- | --- | --- |
|  |  | AUROC | MCC | F1 | Precision | Recall | AUROC | MCC | F1 | Precision | Recall |
| Only Classical  Sequence Derived  Features | LR | 0.836 | 0.516 | 0.751 | 0.771 | 0.733 | 0.813 | 0.501 | 0.754 | 0.739 | 0.770 |
|  | SVM | 0.872 | 0.612 | 0.814 | 0.772 | 0.863 | 0.851 | 0.613 | 0.818 | 0.740 | 0.914 |
|  | RF | 0.879 | 0.621 | 0.818 | 0.779 | 0.862 | 0.865 | 0.577 | 0.802 | 0.729 | 0.892 |
|  | NN | 0.871 | 0.580 | 0.788 | 0.794 | 0.782 | 0.840 | 0.558 | 0.789 | 0.745 | 0.838 |
|  | FM | 0.882 | 0.616 | 0.812 | 0.805 | 0.858 | 0.875 | 0.612 | 0.815 | 0.765 | 0.872 |
| Only Deep ESM  Embedding  Features | LR | 0.947 | 0.772 | 0.887 | 0.880 | 0.894 | 0.950 | 0.774 | 0.888 | 0.875 | 0.900 |
|  | SVM | 0.917 | 0.695 | 0.850 | 0.833 | 0.869 | 0.909 | 0.706 | 0.855 | 0.838 | 0.872 |
|  | RF | 0.954 | 0.806 | 0.905 | 0.863 | 0.952 | 0.942 | 0.742 | 0.875 | 0.814 | 0.947 |
|  | NN | 0.965 | 0.835 | 0.916 | 0.924 | 0.909 | 0.971 | 0.842 | 0.919 | 0.930 | 0.909 |
|  | FM | 0.968 | 0.840 | 0.921 | 0.919 | 0.916 | 0.970 | 0.846 | 0.922 | 0.924 | 0.920 |
| Integrated  Classical + Deep Embeddings Features | LR | 0.926 | 0.727 | 0.862 | 0.868 | 0.857 | 0.915 | 0.690 | 0.846 | 0.836 | 0.856 |
|  | SVM | 0.933 | 0.743 | 0.875 | 0.841 | 0.913 | 0.914 | 0.712 | 0.862 | 0.798 | 0.936 |
|  | RF | 0.951 | 0.782 | 0.894 | 0.849 | 0.945 | 0.942 | 0.727 | 0.868 | 0.796 | 0.956 |
|  | NN | 0.941 | 0.766 | 0.883 | 0.881 | 0.885 | 0.936 | 0.741 | 0.872 | 0.857 | 0.886 |
|  | FM | 0.960 | 0.773 | 0.894 | 0.878 | 0.921 | 0.951 | 0.760 | 0.883 | 0.850 | 0.918 |

**Table S3.** Comparative Performance of AMR Prediction Methods on the Held-Out Test dataset, in terms of AUROC, MCC, F1-Score, Precision, and Recall metrics.

| Method | Strategy | AUROC | MCC | F1 | Precision | Recall |
| --- | --- | --- | --- | --- | --- | --- |
| Classical-LR | alignment-free, classical features | 0.817 | 0.505 | 0.759 | 0.743 | 0.777 |
| Classical-SVM | alignment-free, classical features | 0.827 | 0.533 | 0.785 | 0.718 | 0.864 |
| Classical-RF | alignment-free, classical features | 0.826 | 0.504 | 0.771 | 0.710 | 0.842 |
| Classical-NN | alignment-free, classical features | 0.840 | 0.519 | 0.769 | 0.743 | 0.796 |
| Classical-FM | alignment-free, classical features | **0.850** | **0.568** | **0.799** | **0.738** | **0.871** |
| Deep-LR | alignment-free, deep embeddings | 0.935 | 0.727 | 0.866 | 0.855 | 0.877 |
| Deep-SVM | alignment-free, deep embeddings | 0.894 | 0.673 | 0.842 | 0.818 | 0.867 |
| Deep-RF | alignment-free, deep embeddings | 0.942 | 0.751 | 0.881 | 0.823 | 0.927 |
| Deep-NN | alignment-free, deep embeddings | 0.963 | 0.818 | 0.911 | 0.893 | 0.930 |
| Deep-FM | alignment-free, deep embeddings | **0.966** | **0.818** | **0.911** | **0.891** | **0.932** |
| Integrated-LR | alignment-free, integrated features | 0.919 | 0.694 | 0.849 | 0.844 | 0.853 |
| Integrated-SVM | alignment-free, integrated features | 0.904 | 0.661 | 0.840 | 0.780 | 0.910 |
| Integrated-RF | alignment-free, integrated features | 0.934 | 0.713 | 0.864 | 0.796 | 0.945 |
| Integrated-NN | alignment-free, integrated features | 0.936 | 0.734 | 0.870 | 0.854 | 0.886 |
| Integrated-FM | alignment-free, integrated features | **0.943** | **0.765** | **0.887** | **0.853** | **0.923** |

**Table S4.** A summary of model stability and error bounds across randomized seed initializations. Metrics report the mean value ± standard deviation across 10 independent iterations with fully randomized data partitioning, hyperparameter tuning, and weight initialization states for test dataset.

| Model | Val AUROC | Test AUROC | Val MCC | Test MCC | Val F1 | Test F1 | Val Precision | Test Precision | Val Recall | Test Recall |
| --- | --- | --- | --- | --- | --- | --- | --- | --- | --- | --- |
| Classical-LR | 0.8113 ± 0.0001 | 0.8119 ± 0.0001 | 0.5083 ± 0.0019 | 0.4806 ± 0.0036 | 0.7561 ± 0.0011 | 0.7436 ± 0.0014 | 0.7458 ± 0.0014 | 0.7391 ± 0.0027 | 0.7666 ± 0.0026 | 0.7481 ± 0.0016 |
| Classical-SVM | 0.8586 ± 0.0000 | 0.8442 ± 0.0000 | 0.6116 ± 0.0014 | 0.5572 ± 0.0012 | 0.8171 ± 0.0006 | 0.7939 ± 0.0006 | 0.7449 ± 0.0007 | 0.7352 ± 0.0002 | 0.9046 ± 0.0016 | 0.8628 ± 0.0011 |
| Classical-RF | 0.8681 ± 0.0031 | 0.8330 ± 0.0027 | 0.6111 ± 0.0141 | 0.5120 ± 0.0130 | 0.8172 ± 0.0061 | 0.7787 ± 0.0048 | 0.7221 ± 0.0152 | 0.6928 ± 0.0131 | 0.9420 ± 0.0191 | 0.8895 ± 0.0173 |
| Classical-NN | 0.8369 ± 0.0110 | 0.8358 ± 0.0090 | 0.5589 ± 0.0189 | 0.5267 ± 0.0149 | 0.7891 ± 0.0119 | 0.7766 ± 0.0123 | 0.7424 ± 0.0138 | 0.7313 ± 0.0159 | 0.8434 ± 0.0355 | 0.8300 ± 0.0445 |
| Classical-FM | 0.8673± 0.0042 | 0.8511 ± 0.0008 | 0.6208 ± 0.0126 | 0.5722 ± 0.0105 | 0.8122 ± 0.0072 | 0.8006 ± 0.0037 | 0.7484 ± 0.0023 | 0.7403 ± 0.0013 | 0.9355 ± 0.0134 | 0.8815 ± 0.0145 |
| Deep-LR | 0.9374 ± 0.0001 | 0.9389 ± 0.0000 | 0.7651 ± 0.0000 | 0.7419 ± 0.0025 | 0.8794 ± 0.0000 | 0.8681 ± 0.0010 | 0.8966 ± 0.0000 | 0.8905 ± 0.0022 | 0.8628 ± 0.0000 | 0.8468 ± 0.0000 |
| Deep-SVM | 0.9787 ± 0.0000 | 0.9655 ± 0.0000 | 0.8801 ± 0.0000 | 0.8388 ± 0.0011 | 0.9407 ± 0.0000 | 0.9218 ± 0.0005 | 0.9179 ± 0.0000 | 0.8860 ± 0.0009 | 0.9646 ± 0.0000 | 0.9606 ± 0.0000 |
| Deep-RF | 0.9489 ± 0.0010 | 0.9418 ± 0.0023 | 0.7869 ± 0.0063 | 0.7560 ± 0.0090 | 0.8954 ± 0.0034 | 0.8823 ± 0.0044 | 0.8653 ± 0.0119 | 0.8490 ± 0.0106 | 0.9281 ± 0.0159 | 0.9186 ± 0.0145 |
| Deep-NN | 0.9727 ± 0.0038 | 0.9625 ± 0.0028 | 0.8462 ± 0.0183 | 0.8177 ± 0.0172 | 0.9236 ± 0.0083 | 0.9113 ± 0.0084 | 0.9057 ± 0.0273 | 0.8806 ± 0.0195 | 0.9431 ± 0.0185 | 0.9449 ± 0.0222 |
| Deep-FM | 0.9767± 0.0022 | 0.9751 ± 0.0016 | 0.8913 ± 0.0016 | 0.8519 ± 0.0015 | 0.9433 ± 0.0038 | 0.9276 ± 0.0065 | 0.9214 ± 0.0032 | 0.8953 ± 0.0105 | 0.9685 ± 0.0094 | 0.9698 ± 0.0011 |
| Integrated- LR | 0.9128 ± 0.0001 | 0.9193 ± 0.0001 | 0.7054 ± 0.0017 | 0.6889 ± 0.0032 | 0.8573 ± 0.0008 | 0.8510 ± 0.0014 | 0.8177 ± 0.0019 | 0.8151 ± 0.0030 | 0.9009 ± 0.0020 | 0.8902 ± 0.0031 |
| Integrated- SVM | 0.9270 ± 0.0000 | 0.9207 ± 0.0000 | 0.7400 ± 0.0010 | 0.7070 ± 0.0011 | 0.8744 ± 0.0005 | 0.8604 ± 0.0004 | 0.8203 ± 0.0010 | 0.8141 ± 0.0023 | 0.9361 ± 0.0007 | 0.9123 ± 0.0026 |
| Integrated- RF | 0.9499 ± 0.0015 | 0.9360 ± 0.0029 | 0.7736 ± 0.0059 | 0.7255 ± 0.0077 | 0.8886 ± 0.0034 | 0.8673 ± 0.0040 | 0.8619 ± 0.0160 | 0.8380 ± 0.0132 | 0.9177 ± 0.0216 | 0.8993 ± 0.0179 |
| Integrated- NN | 0.9281 ± 0.0064 | 0.9237 ± 0.0070 | 0.7384 ± 0.0156 | 0.7058 ± 0.0270 | 0.8717 ± 0.0087 | 0.8574 ± 0.0153 | 0.8400 ± 0.0201 | 0.8259 ± 0.0180 | 0.9073 ± 0.0323 | 0.8932 ± 0.0436 |
| Integrated- FM | 0.9517± 0.0026 | 0.9355 ± 0.0113 | 0.7785 ± 0.0095 | 0.7314 ± 0.0105 | 0.9011 ± 0.0043 | 0.8760 ± 0.0057 | 0.8744 ± 0.0029 | 0.8253 ± 0.0117 | 0.9425 ± 0.0092 | 0.9015 ± 0.0121 |

**Table S5.** Computational robustness and generalizability profiles of machine learning architecture across fifty repeated randomized partitions. Performance metrics (reported as empirical Mean ± Standard Deviation) were gathered from the independent out-of-sample test splits (15%) allocation) across fifty unique Monte Carlo shuffling iterations using a fixed 70:15:15 data-splitting ratio. This extensive resampling validation was executed utilizing the Integrated Feature Space, which concatenates high-dimensional protein language model deep embeddings with classical sequence-derived biophysical descriptors to construct a unified feature matrix.

| Classifier Model | Accuracy | Precision | Recall | F1 | MCC |
| --- | --- | --- | --- | --- | --- |
| LR | 0.848 ± 0.0116 | 0.842 ± 0.0268 | 0.863 ± 0.0347 | 0.851 ± 0.0130 | 0.698 ± 0.0224 |
| SVM | 0.867 ± 0.0127 | 0.846 ± 0.0238 | 0.901 ± 0.0324 | 0.872 ± 0.0137 | 0.736 ± 0.0248 |
| RF | 0.887 ± 0.0112 | 0.860 ± 0.0225 | 0.927 ± 0.0236 | 0.892 ± 0.0112 | 0.777 ± 0.0214 |
| NN | 0.858 ± 0.0130 | 0.836 ± 0.0280 | 0.896 ± 0.0384 | 0.864 ± 0.0131 | 0.719 ± 0.0248 |
| FM | 0.886 ± 0.0123 | 0.868 ± 0.0216 | 0.931 ± 0.0316 | 0.885 ± 0.0124 | 0. 778 ± 0.0237 |

**Table S6.** Comprehensive biophysical and AlphaFold3 structural parameters of persistently misclassified AMR protein candidates. Metrics provide a detailed breakdown of sequence properties, regional disorder profiles, structure prediction confidence scores (pLDDT), and secondary structure fractions for the 17 recalcitrant false-negative and false-positive variants, matching the node string entries displayed in Figure 4.

| ID | Mean Disorder Score | % Disordered Residues | Helices | Strands | Coils | pLDDT |
| --- | --- | --- | --- | --- | --- | --- |
| AMRF_162 | 0.07 | 0.00 | 0.47 | 0.17 | 0.36 | 87.6 |
| AMRF_219 | 0.11 | 3.48 | 0.58 | 0.13 | 0.29 | 79.4 |
| AMRF_2499 | 0.05 | 0.00 | 0.19 | 0.26 | 0.55 | 88.2 |
| AMRF_2603 | 0.08 | 3.40 | 0.51 | 0.15 | 0.35 | 85.5 |
| AMRF_2661 | 0.12 | 9.46 | 0.07 | 0.40 | 0.53 | 86.8 |
| AMRF_2692 | 0.11 | 4.28 | 0.52 | 0.15 | 0.33 | 84.2 |
| AMRF_450 | 0.22 | 17.53 | 0.36 | 0.31 | 0.33 | 77.3 |
| AMRF_527 | 0.16 | 5.42 | 0.78 | 0.00 | 0.22 | 76.5 |
| AMRF_549 | 0.07 | 2.10 | 0.75 | 0.05 | 0.19 | 86.9 |
| AMRF_562 | 0.36 | 20.27 | 0.99 | 0.00 | 0.01 | 76.0 |
| AMRF_589 | 0.04 | 0.00 | 0.83 | 0.00 | 0.17 | 90.7 |
| CARD_685 | 0.11 | 3.42 | 0.53 | 0.15 | 0.33 | 84.1 |
| CARD_853 | 0.04 | 0.00 | 0.34 | 0.25 | 0.41 | 90.5 |
| NAMR_13919 | 0.15 | 1.62 | 0.47 | 0.16 | 0.36 | 78.4 |
| NAMR_15458 | 0.15 | 7.89 | 0.05 | 0.45 | 0.50 | 76.0 |
| NAMR_16364 | 0.08 | 2.05 | 0.84 | 0.00 | 0.16 | 85.8 |
| NAMR_17406 | 0.56 | 53.08 | 0.53 | 0.00 | 0.47 | 52.3 |

**Table S7.** Model classification performance on the external evaluation dataset (n = 527)**.** Metrics evaluate the generalizability of pre-trained models on a novel cohort consisting of β-lactamases (n = 272) balanced against diverse non-AMR RefSeq bacterial sequences (n = 255), executed without any model re-training or weight adjustments.

| Method | AUROC | MCC | F1 | Precision | Recall |
| --- | --- | --- | --- | --- | --- |
| Classical-LR | 0.7698 | 0.5453 | 0.7611 | 0.7945 | 0.8177 |
| Classical-SVM | 0.7834 | 0.5065 | 0.7243 | 0.7384 | 0.7588 |
| Classical-RF | 0.7895 | 0.5274 | 0.6922 | 0.6839 | 0.7654 |
| Classical-NN | 0.7755 | 0.5612 | 0.8037 | 0.7464 | 0.8410 |
| Classical-FM | 0.8325 | 0.5867 | 0.8150 | 0.6903 | 0.7654 |
| Deep-LR | 0.9491 | 0.7220 | 0.8084 | 0.8376 | 0.8784 |
| Deep-SVM | 0.9250 | 0.7313 | 0.8167 | 0.8065 | 0.8602 |
| Deep-RF | 0.9632 | 0.7768 | 0.8841 | 0.8284 | 0.8968 |
| Deep-NN | 0.9717 | 0.7883 | 0.8641 | 0.9104 | 0.9408 |
| Deep-FM | 0.9765 | 0.7596 | 0.8919 | 0.9123 | 0.9255 |
| Integrated-LR | 0.9321 | 0.6439 | 0.8556 | 0.8575 | 0.8858 |
| Integrated-SVM | 0.9193 | 0.6641 | 0.8695 | 0.8071 | 0.8510 |
| Integrated-RF | 0.9528 | 0.7265 | 0.9092 | 0.8216 | 0.8928 |
| Integrated-NN | 0.9484 | 0.7607 | 0.8672 | 0.8706 | 0.8871 |
| Integrated-FM | 0.9534 | 0.7727 | 0.8917 | 0.8812 | 0.8923 |

**SUPPLEMENTARY FIGURES**

**Figure S1.** Multi-panel 3×3 grid representation of filtered principal component subspaces. Columns correspond to isolated cumulative variance targets (50%, 80%, and 95% from left to right) reconstructed from standardized feature spaces. Rows display the independent coordinate distributions for Classical Descriptors (top row), Deep ESM Embeddings (middle row), and the Integrated Hybrid Space (bottom row). Red data markers denote antimicrobial resistance (AMR) proteins, while blue markers represent Non-AMR sequences. Due to high dimensional complexity, class marker overlap is expected on flat 2D planes, while information density differences are captured by the primary variance capture ratios. At the 95% variance checkpoint, the Classical and Integrated subspace coordinate axes are diluted down to capturing minimal localized variance (Classical PC1: 0.7%, PC2: 0.4%; Integrated PC1: 1.7%, PC2: 1.1%), whereas the Deep ESM space maintains a concentrated information density along its primary components (PC1: 9.1%, PC2: 6.7%).

**Figure S2.** Standardized Volcano Plot mapping feature importance and architectural scaling profiles. The vertical axis tracks statistical significance (-log_10_ Adjusted *p*-value) derived from non-parametric two-sided Mann-Whitney U tests with Benjamini-Hochberg False Discovery Rate (FDR) corrections. The horizontal axis maps the Standardized Effect Size (Cohen’s d) to resolve scale mismatches between distinct representational spaces. Blue circles represent Deep ESM Embeddings, which demonstrate high statistical significance scaling intensely up to the 10^-100^ range across a balanced effect spectrum. Red triangles track Classical Descriptors, which contain informative signals but operate on highly diffused, multi-collinear unscaled geometric ranges. The dashed horizontal line marks the strict significance threshold (FDR = 0.05). Key structural anchors (such as sequence length, molecular weight, pI, and instability index) are highlighted with explicit directional annotations to illustrate the scalar differences that introduce multi-collinear noise when directly appended into an integrated hybrid space.
